# Impact of dietary vitamin D on immunoregulation and disease pathology in lupus-prone NZB/W F1 mice

**DOI:** 10.3389/fimmu.2022.933191

**Published:** 2022-11-24

**Authors:** Antoine N. Kraemer, Anna-Lena Schäfer, Dalina T. L. Sprenger, Bettina Sehnert, Johanna P. Williams, Aileen Luo, Laura Riechert, Qusai Al-Kayyal, Hélène Dumortier, Jean-Daniel Fauny, Zoltan Winter, Kathrin Heim, Maike Hofmann, Martin Herrmann, Guido Heine, Reinhard E. Voll, Nina Chevalier

**Affiliations:** ^1^ Department of Rheumatology and Clinical Immunology, Medical Center - University of Freiburg, Faculty of Medicine, University of Freiburg, Freiburg, Germany; ^2^ Center for Chronic Immunodeficiency (CCI), Medical Center - University of Freiburg, Faculty of Medicine, University of Freiburg, Freiburg, Germany; ^3^ Centre national de la recherche scientifique (CNRS) UPR3572, Immunology, Immunopathology and Therapeutic Chemistry, Institute of Molecular and Cellular Biology, Strasbourg, France; ^4^ Institute of Radiology, Preclinical Imaging Platform Erlangen (PIPE), Friedrich-Alexander-University Erlangen-Nürnberg, Erlangen, Germany; ^5^ Department of Gastroenterology, Hepatology, Endocrinology and Infectious Diseases, Medical Center - University of Freiburg, Faculty of Medicine, University of Freiburg, Freiburg, Germany; ^6^ Department of Internal Medicine 3, and Deutsches Zentrum Immuntherapie (DZI), University Medical Center Erlangen, Friedrich-Alexander-University Erlangen-Nürnberg, Erlangen, Germany; ^7^ Division of Allergy, University Hospital Schleswig-Holstein, Kiel, Germany

**Keywords:** vitamin D, autoimmunity, lupus, SLE, diet, choleacliferol, mice, autoimmune

## Abstract

Vitamin D (VD) deficiency is a highly prevalent worldwide phenomenon and is extensively discussed as a risk factor for the development of systemic lupus erythematosus (SLE) and other immune-mediated diseases. In addition, it is now appreciated that VD possesses multiple immunomodulatory effects. This study aims to explore the impact of dietary VD intake on lupus manifestation and pathology in lupus-prone NZB/W F1 mice and identify the underlying immunological mechanisms modulated by VD. Here, we show that low VD intake accelerates lupus progression, reflected in reduced overall survival and an earlier onset of proteinuria, as well higher concentrations of anti-double-stranded DNA autoantibodies. This unfavorable effect gained statistical significance with additional low maternal VD intake during the prenatal period. Among examined immunological effects, we found that low VD intake consistently hampered the adoption of a regulatory phenotype in lymphocytes, significantly reducing both IL-10-expressing and regulatory CD4^+^ T cells. This goes along with a mildly decreased frequency of IL-10-expressing B cells. We did not observe consistent effects on the phenotype and function of innate immune cells, including cytokine production, costimulatory molecule expression, and phagocytic capacity. Hence, our study reveals that low VD intake promotes lupus pathology, likely *via* the deviation of adaptive immunity, and suggests that the correction of VD deficiency might not only exert beneficial functions by preventing osteoporosis but also serve as an important module in prophylaxis and as an add-on in the treatment of lupus and possibly other immune-mediated diseases. Further research is required to determine the most appropriate dosage, as too-high VD serum levels may also induce adverse effects, possibly also on lupus pathology.

## Introduction

Systemic lupus erythematosus (SLE) is a chronic autoimmune disease affecting many organs and causing significant morbidity and mortality. SLE is characterized by immune dysregulation and a breakdown of tolerance to self-antigens. This results in the production of autoantibodies, which are most commonly directed against nuclear self-antigens, in particular double-stranded DNA (dsDNA). These autoantibodies may form immune complexes (ICs) with autoantigens, ultimately leading to inflammation and tissue damage (1, 2). In SLE, the kidneys are often afflicted by this inflammation, which can result in lupus nephritis (LN), which eventually leads to renal failure (3).

Generally, in SLE, there may be defects in various branches of the immune system, explaining the great heterogeneity of clinical presentations. Several dysregulations within the innate immune compartment have been reported in the context of SLE, including an impaired clearance of apoptotic debris by macrophages. For instance, in the germinal centers (GCs) of the lymph nodes, this results in an increased exposure of nuclear self-antigens and increases the risk of autoreactive B-cell activation by follicular dendritic cells and ultimately drives the production of autoantibodies (4–7). Furthermore, monocytes and macrophages in SLE display abnormal cytokine production, adopting a more pro-inflammatory cytokine profile (8–10). The adaptive immune system is also afflicted by dysregulation in patients with SLE. For instance, studies have reported an increased presence of B-cell survival factors that increase the activation and survival of autoreactive B cells (11, 12). In addition, an increase in follicular T helper (T_FH_) cells and a decrease of regulatory T cells (Tregs) has been reported in SLE (13–15).

The observation that autoimmune diseases, such as SLE, are usually rare among non-Westernized populations suggests a significant impact of the profound lifestyle and environmental changes in modern Western societies on the development of such diseases. Although the exact pathological mechanisms underlying SLE are still elusive, it is becoming increasingly clear that the pathogenesis of SLE and other immune-mediated diseases are the result of a complex interplay between genetic and environmental factors. The environmental factors that have been suggested to pose a risk for developing SLE include infectious agents, Western dietary habits, and ultraviolet (UV) radiation inducing apoptotic cell death. A highly discussed environmental risk factor for SLE and other autoimmune diseases is the deficiency of vitamin D (VD) (16–20). Multiple studies have shown a high prevalence of VD deficiency among SLE patients, likely, in part, due to the fact that the majority of SLE patients are hypersensitive to disease-triggering UV light and thus protect themselves from sunlight (21–29). However, the relevance of VD on the pathogenesis, whether it is a secondary phenomenon or involved in disease manifestation and progression, is not entirely clear.

VD is a secosteroid hormone and is biosynthesized in the epidermis upon exposure to UV-B radiation, or ingested from dietary sources, such as cod liver oil, and supplements, all in the form of cholecalciferol (vitamin D_3_). VD ultimately exerts its effects *via* the regulation of genes and the epigenome, through the actions of the active VD metabolite 1,25(OH)_2_D_3_ (calcitriol) binding the VD receptor, which is expressed by many immune cells (30–35). Although VD is most prominently known for its regulatory role in calcium and phosphate homeostasis, the immunomodulatory effects of VD have become increasingly appreciated. For instance, VD promotes the differentiation of monocytes into macrophages while inhibiting the maturation of differentiating dendritic cells (DCs) (36–39). In the latter, VD induces a tolerogenic phenotype by suppressing their antigen-presenting capacity, thus inhibiting the ability to activate T cells (39–41). In the adaptive immune system, VD is known to promote Treg differentiation while inhibiting T_H_1 and T_H_17 cell differentiation (42–46). There is also *in vitro* evidence showing that VD inhibits the differentiation of B cells into plasma cells, decreases antibody production, and promotes IL-10 expression (47–51).

The ability of VD to regulate antigen presentation and tip lymphocyte differentiation toward a more anti-inflammatory or immunoregulatory phenotype suggests therapeutic potential for SLE and other autoimmune diseases. Therefore, we investigated the effects of dietary VD intake on lupus pathology and associated immunological effects in NZB/W F1 mice, a spontaneous model of murine lupus (52). We found that low VD intake accelerates lupus progression, with this effect becoming significant when low VD intake commenced during the prenatal period. In our broad immune-phenotypic analysis, we found that low VD intake consistently hampered the adoption of a regulatory phenotype in lymphocytes. Hence, our data provide evidence of possible adverse effects of VD deficiency on lupus progression and encourage further studies to elucidate detailed mechanisms. These should also address optimal dosage, as excessively elevated VD serum levels may exert adverse effects, possibly also on lupus progression.

## Materials and methods

### Mice and models

Lupus-prone NZB/W F1 mice were generated by crossing NZB/BlNJ with NZW/LacJ mice, which were purchased from *The Jackson Laboratory*. CD45.1 C57BL/6 animals (B6.SJL-*Ptprc^a^ Pepc^b^*/BoyJ) were bred in our own facility. For all experiments, the mice were housed in individually ventilated cages on a 12 h light/dark cycle, with food and water *ad libitum*. To test the influence of VD, randomized mice were fed the following diets: a low-VD diet (< 50 IU/kg cholecalciferol and Ca:P=2.5:1, *Altromin C 1017 mod.*), a normal VD diet (500 IU/kg cholecalciferol and Ca:P=1.2:1, *Altromin C 1000 control diet*) or a high-VD diet (76,500 or 38,000 IU/kg cholecalciferol and Ca:P=1.5:1, Altromin C 1017 mod.). All diets were purchased from *Altromin*. In a first VD-feeding approach, treatments were started after weaning at roughly 5 weeks of age and continued throughout the entire duration of the experiment. In a second VD-feeding approach, NZB/BlNJ and NZW/LacJ parent animals were also fed the different VD diets and NZB/W F1 offspring were weaned on the same food that was fed continuously throughout the entire duration of the experiment. Blood and urine were collected and mice were euthanized at defined time points for organ harvest and downstream experiments. Animals were regularly monitored and euthanized when reaching defined ethical endpoints [proteinuria in addition to a deteriorating general health condition and/or significant weight loss (≥ 20% over the course of 2 days)].

### Inverted screen test

To determine grip strength, mice were placed individually on top of the center of a stainless-steel grid (50 cm × 30 cm) with a mesh size of 12 mm and a thickness of 1.6 mm. The grid was subsequently inverted by 180°, resulting in mice hanging upside down on all four limbs roughly 30 cm over a soft landing spot. The duration of hanging time was measured and normalized to bodyweight.

### Measurement of bone density

For the µCT scanning of the tibiae, a dedicated preclinical scanner (Siemens Inveon PET/CT/SPECT Multimodality System, Siemens Healthineers, Germany) was used at a tube voltage of 80 kV and a tube current of 500 μA. The samples were positioned, fixed, and images were acquired with an isotropic resolution of 17.04 μm.

### Assessment of proteinuria

Urine samples were collected by spontaneous urination. For a semiquantitative measurement of proteinuria, Albustix test strips (*Siemens*) were used. According to the color scale provided by the manufacturer, albuminuria was categorized as follows: 0–1 = trace, 1 = 30 mg/dl, 2 = 100 mg/dl, 3 = 300 mg/dl, and 4 > 2,000 mg/dl. Mice were deemed proteinuric after scoring a 2 on the color scale for at least two consecutive weeks.

### Assessment of anti-double-stranded DNA Immunoglobulin G (IgG) autoantibodies and total IgG antibodies

Serum titers of IgG autoantibodies directed against dsDNA, as well as serum titers of total IgG were determined by enzyme-linked immunosorbent assay (ELISA). For anti-dsDNA IgG ELISAs, 384-well microtiter plates (*Greiner Bio One*) were precoated with 15 µl 20 µg/ml Poly-L-Lysin (*Sigma-Aldrich*) for 1 h at 37°C, followed by coating with 15 µl 20 µg/ml calf thymus DNA (*Sigma-Aldrich*) at 4°C overnight (o/n). Plates were blocked with 2% fetal calf serum (FCS) in Phosphate-buffered saline (PBS) for 2 h at RT. For total IgG ELISAs, 384-well microtiter plates were coated with 15 µl 1 µg/ml goat anti-mouse IgG (*Jackson ImmunoResearch*) at 4°C o/n. The plates were blocked with 2% FCS in PBS for 2 h at RT. Samples were diluted in 2% FCS in PBS and 40 µl incubated for 2 h at RT. Bound IgG was detected with 15 µl 160 ng/µl HRP-conjugated goat anti-mouse IgG secondary antibody (*Jackson ImmunoResearch*). Development was performed with a 3,3’5,5’-Tetramethylbenzidine (TMB) substrate (*Thermo Fisher Scientific*), according to the manufacturer’s protocol. The absorbance at 450 nm was measured using the Spark^®^ 10 M multimode microplate reader (*Tecan*). To determine anti-dsDNA IgG titers, expressed as arbitrary units (A.U.), reference sera of nephritic female NZB/W F1 mice were used to create a standard curve. To determine total IgG titers, expressed as A.U., reference sera of wild-type C57BL/6 mice were used to create a standard curve.

### Flow cytometry

Single-cell suspensions of the spleen were obtained by the mechanic dissociation of the spleen and of the bone marrow by flushing out the bone marrow from truncated tibiae and femurs *via* centrifugation. Peritoneal exudate cells (PECs) were obtained *via* peritoneal lavage; peripheral blood immune cells were retrieved from blood collected in Ethylenediaminetetraacetic acid (EDTA)-coated tubes. Red blood cell (RBC) lysis was performed for 5 min, for all cell suspensions, except PECs. Following incubation with anti-CD16/32 antibodies (*101330*, *BioLegend*) to block non-specific Fc receptor binding, single-cell suspensions were stained with Biotin- or fluorochrome-conjugated monoclonal antibodies diluted in 2% FCS/PBS for 30 min on ice. For intracellular and intranuclear staining, cells were fixed and permeabilized with BD Cytofix/Cytoperm (*BD Biosciences*) and the eBioscience FoxP3/Transcription Factor Staining Buffer Set (*eBioscience*), respectively. For intracellular cytokine staining, cells were stimulated with 50 ng/ml PMA (*Sigma-Aldrich*), 1 µg/ml ionomycin (*Sigma-Aldrich*), and brefeldin A (*eBioscience*) for 4 h at 37°C/5% CO_2_ prior to staining and fixation. To identify apoptotic cells, annexin V staining was performed using Annexin V Binding Buffer (*BD Biosciences*). The following anti-mouse antibodies were used: B220/CD45R Biotin (*103204, BioLegend*), B220/CD45R Pacific Blue (*103230, BioLegend*), B220/CD45R APC-Cy7 (*103224, BioLegend*), CD11b Biotin (*13-0112-82, eBioscience*), CD11b FITC (*101206, BioLegend*), CD11b PE-Cy7 (*101215, BioLegend*), CD11c APC (*117309, BioLegend*), CD11c Biotin (*117303, BioLegend*), CD11c PE-Cy7 (*117318, BioLegend*), CD127/IL-7Rα PE-Cy7 (*135014, BioLegend*), CD138 BV421 (*142508, BioLegend*), CD16/32 (*101330, BioLegend*), CD16/32 FITC (*MCA2305F, Serotec*), CD19 Biotin (*115504, BioLegend*), CD200R PE (*123907, BioLegend*), CD206 APC (*141707, BioLegend*), CD2 Biotin (*100103, BioLegend*), CD34 APC (*128606, BioLegend*), CD301 PE-Cy7 (*145705, BioLegend*), CD36 APC/Fire 750 (*102617, BioLegend*), CD3ϵ Biotin (*13-0031-85, eBioscience*), CD4 Biotin (*100404, BioLegend*), CD4 PE-Cy7 (*100421, BioLegend*), CD45 eFluor506 (*69-0451-82, eBioscience*), CD45.1 APC (*110714, BioLegend*), CD45.2 Pacific Blue (*109819, BioLegend*), CD80 APC/Fire 750 (*104739, BioLegend*), CD86 APC (*105011, BioLegend*), CD8α PerCP (*100732, BioLegend*), CD8β PerCP-Cy5.5 (*140417, BioLegend*), c-kit/CD117 PE (*553355, BD Pharmingen*), CXCR5 Biotin (*551960, BD Pharmingen*), F4/80 APC-Cy7 (*123117, BioLegend*), F4/80 FITC (*123107, BioLegend*), Fas/CD95 PE (*554258, BD Pharmingen*), FoxP3 APC (*14-5773-82, eBioscience*), GL7 FITC (*553666, BD Pharmingen*), I-A/I-E Pacific Blue (*107619, BioLegend*), ICOS-L PE (*107405, BioLegend*), IFN-γ APC (*505809, BioLegend*), IL-10 FITC (*505006, BioLegend*), IL-17A PE (*559502, BD Pharmingen*), Ly6C PE-Cy7 (*560593, BD Pharmingen*), Ly6G Biotin (*127604 BioLegend*), Ly6G Pacific Blue (*127611, BioLegend*), MerTK PE (*151505, BioLegend*), NK1.1 APC-Cy7 (*108723, BioLegend*), NK1.1 Biotin (*108704, BioLegend*), PD-1/CD279 PE (*12-9985-83, eBioscience*), PDCA-1/CD317 PE (*12-3172-82 eBioscience*), Sca-1/Ly6A/E Pacific Blue (*108120, BioLegend*), TACI/CD267 APC (*17-5942-81, eBioscience*), TCR-β APC-Cy7 (*109220, BioLegend*), TCR-β Biotin (*109204, BioLegend*), TCR-β PerCP-Cy5.5 (*109227, BioLegend*), TER-119 Biotin (*116203, BioLegend*), Tim-4 PE-Cy7 (*130009, BioLegend*).

As described previously (53), the following immune cell subsets were identified: B cells (% TCR-β^-^B220^+^/single cells), germinal center B cells (GC B cells) (% Fas^hi^GL7^hi^/B cells), CD4^+^ and CD8^+^ T cells (% TCR-β^+^B220^-^CD4^+^CD8^-^ or TCR-β^+^B220^-^CD4^-^CD8^+^/single cells), expression of IL-10 in B cells, as well as IL-10, IL-17A and IFN-γ in CD4^+^ or CD8^+^ T cells, regulatory T cells (Tregs) (% FoxP3^+^/CD4^+^ T cells), follicular T helper (T_FH_) cells (% CXCR5^hi^PD-1^hi^/CD4^+^ T cells), conventional dendritic cells (cDCs) and plasmacytoid dendritic cells (pDCs) (% CD11c^hi^PDCA-1^-^CD19^-^TCR-β^-^NK1.1^-^ or PDCA-1^hi^CD19^-^TCR-β^-^NK1.1^-^/single cells), CD11b^+^ monocytic cells (% CD19^-^TCR-β^-^NK1.1^-^PDCA-1^-^CD11c^-^CD11b^+^Ly6G^-^/single cells), circulating CD11b^+^ monocytic cells (% Ly6G^-^CD11b^+^/CD45^+^ cells), neutrophils (%CD19^-^TCR-β^-^NK1.1^-^PDCA-1^-^CD11c^-^CD11b^+^Ly6G^hi^/single cells), circulating neutrophils (% CD11b^+^ Ly6G^hi^/CD45^+^ cells), natural killer (NK) cells (% CD19^-^TCR-β^-^NK1.1^+^/single cells) and expression of CD80 and CD86 on CD11b^+^ monocytic cells, cDCs, peritoneal macrophages, and B cells (**Supplementary Figures 11, 12**). Further immune cell subsets were also identified: plasma cells (PCs) (% CD138^hi^/single cells); peritoneal macrophages (F4/80^+^Ly6G^int/-^); Ly6C^hi^ peritoneal macrophages (% Ly6C^hi^/peritoneal macrophages); Ly6C^hi^ CD11b^+^ monocytic cells (% Ly6C^hi^/CD11b^+^ monocytic cells); the expression of ICOS-L on CD11b^+^ monocytic cells, cDCs, peritoneal macrophages and B cells; and the expression of Tim-4, MerTK, CD206, I-A/I-E, CD36, CD301 and CD200R on peritoneal macrophages and bone marrow–derived macrophages (BMDMs). In peripheral blood, additional CD45-staining was included to identify leukocytes. Flow cytometric analysis was performed at the BD LSR Fortessa flow cytometer (*Becton Dickinson*) followed by data analysis using FlowJo™ Software (*Becton Dickinson).*

### Fluorescence-activated cell sorting of splenic CD11b^+^ innate immune cells and peritoneal macrophages

Single-cell suspensions of splenocytes were obtained by mechanic dissociation of the spleen, while the single-cell suspensions of PECs were obtained following peritoneal lavage. RBC lysis was performed for 5 min for the single-cell suspensions of splenocytes. Cells were subsequently stained with a cocktail of antibodies. The following anti-mouse antibodies were used: CD11b FITC (*101206, BioLegend*), CD11b PE (*101207, BioLegend*), CD11c APC (*117309, BioLegend*), CD19 FITC (*115505, BioLegend*), F4/80 PE (*123109, BioLegend*), Ly6G Pacific Blue (*127611, BioLegend*), NK1.1 FITC (*108705, BioLegend*), and TCR-β FITC (*109205, BioLegend*). Splenic CD11b^+^ innate immune cells were sorted as CD19^-^TCR-β^-^NK1.1^-^Ly6G^-^CD11b^+^, while peritoneal macrophages were sorted as F4/80^+^CD11c^-^Ly6G^-^CD11b^+^ (**Supplementary Figure 13**). Splenic CD11b^+^ innate immune cells and peritoneal macrophages were sorted by BD FACSMelody™ (*Becton Dickinson*) and stimulated with 50 ng/ml LPS for 2 h 45 min at 37°C/5% CO_2_ followed by RNA isolation.

### Assessment of cytokine secretion by bone marrow–derived innate immune cells and macrophages

0.4 × 10^6^ murine bone marrow (BM) cells were seeded per well in a 48-well culture plate and cultured for 5 days in the presence of 20 ng/ml GM-CSF (*Peprotech*) or 20 ng/ml M-CSF (*Peprotech*) to generate BM-derived innate immune cells and BM-derived macrophages (BMDMs), respectively. The medium supplemented with GM-CSF or M-CSF was refreshed on day 3. On day 5 of incubation and immediately after an additional exchange of the culture medium, cells were stimulated with either 50 ng/ml LPS (*Sigma-Aldrich*) and 50 ng/ml Pam3CSK4 (*In vivogen*) or 1 µM CpG (*In vivogen*) and 1 µM R848 (*Enzo Life Science*) for 20 h. Cytokine concentrations in the culture supernatants were assessed by ELISA. Cytokine concentrations were determined using the IL-10, IL-1β, and TNF-α DuoSet kits (*R&D Systems*), according to the manufacturer’s protocol. The absorbance at 450 nm was measured using the Spark^®^ 10 M multimode microplate reader (*Tecan*). Cytokine concentrations in supernatants were normalized to the total protein content of adherent cells, which was determined *via* BCA assay, according to the manufacturer’s protocol (*Thermo Fisher Scientific*).

### Phagocytosis assay

The phagocytic capacity of both murine peritoneal macrophages and BMDMs (both CD45.2^+^) was investigated, by co-culturing said cells with CD45.1^+^ apoptotic thymocytes (ATs). Thymocytes were isolated from CD45.1^+^ C57BL/6 mice. After mechanical disruption to generate a single-cell suspension, thymocytes were treated with 1 µM dexamethasone for 6 h to induce apoptosis and subsequently labeled with CFDA-SE. 0.5 × 10^6^ PECs were seeded per well in a 96-well U-bottom culture plate and rested for 2 h at 37°C/5% CO_2_. 2.5 × 10^6^ CFDA-SE-labeled ATs were then added to PECs and coincubated for 1 h at 37°C/5% CO_2_. PECs were then placed on ice and washed and stained for flow cytometry in order to identify the percentage of peritoneal macrophages that had phagocytosed ATs. In a second phagocytosis assay, 0.4 × 10^6^ murine BM cells were seeded per well in a 48-well culture plate and cultured for 5 days in the presence of 20 ng/ml M-CSF (*Peprotech*) to generate differentiated adherent BMDMs. The medium supplemented with M-CSF was refreshed on day 3. On day 5 of incubation, 2.5 × 10^6^ ATs were added to BMDMs and coincubated for 1 h at 37°C/5% CO_2_. BMDMs were then washed, detached from the well bottom with trypsin 0.25% (*Anprotec*), and stained for flow cytometry in order to identify the percentage of BMDMs that had phagocytosed ATs.

### Quantitative reverse transcription PCR

Total RNA was extracted from homogenized kidney tissue or purified cells using TRIzol reagent (*Invitrogen*) or the RNeasy^®^ Micro Kit (*Qiagen*), respectively. The QuantiTect Reverse Transcription Kit (*Qiagen*) was used for cDNA synthesis according to the manufacturer’s protocol. Transcripts were quantified by quantitative reverse transcription PCR (RT-qPCR) on a StepOnePlus™ Real-Time PCR System (*Applied Biosystems*) with predesigned TaqMan Gene Expression Assays and reagents according to the manufacturer’s instructions (*Applied Biosystems*), alternatively with predesigned SYBR Green master mixes (*Thermo Fisher Scientific*) and custom-designed primers (**Supplementary Table 1**). Probes with the following Applied Biosystems assay identification numbers were used: Mm99999915_g1 (*GAPDH*), Mm01288386_m1 (*IL1B*), Mm00446190_m1 (*IL6*), and Mm00443258_m1 (*TNF*). For each sample, mRNA abundance was normalized to the amount of GAPDH and is presented as fold gene expression **(2^(ΔΔCt)^).**

### Immunofluorescence

Kidney specimens were embedded in O.C.T. compound (*Tissue-Tek*), snap-frozen on dry ice, and cut into 8 µm sections using a cryotome (Zeiss, Germany). Dried kidney sections were immersed in pre-cooled acetone for 20 min on ice for fixation. Kidney sections were blocked with 10% FCS, 0.3% Triton in PBS for 2 h at RT and stained with FITC-conjugated polyclonal goat anti-mouse IgG (*SouthernBiotech*) overnight at 4°C. After washing with 0.05% Tween 20 in PBS, sections were fixed again in 4% PFA in PBS for 15 min at RT. After four rounds of washing, sections were incubated with DAPI (*Roche*) for 10 min at RT for the labeling of nuclei. Sections were mounted with a fluorescent Mounting Medium (*Dako*) and imaged with a fluorescence microscope (AxioImager 2, *Zeiss*), using a monochrome camera at ×20 magnification. For IgG quantification, the mean intensity of fluorescence in 30 glomeruli per section was determined.

### Blood cell counts

Cell counts, including RBCs, platelets, white blood cells (WBCs), and WBC subtypes, in freshly isolated murine blood were determined by collecting blood in EDTA-coated tubes and analyzing them using a hematology cell counter (Scil Vet abc and Scil Vet abc Plus+).

### Statistical analysis

For statistical analysis, InStat software Prism 9 (*GraphPad software*) was used. *P*-values less than or equal to *0.05* were considered significant. A statistical comparison between two experimental groups was performed using an unpaired t-test (normally distributed data), or a Mann–Whitney U-test (non-parametric data). A statistical comparison between three experimental groups was performed using an ordinary one-way ANOVA (normally distributed data) or a Kruskal–Wallis test (non-parametric data). The Kaplan–Meier method was used for estimating and displaying overall survival (OS) rates and onset of proteinuria. Outliers were determined *via* the Robust regression and outlier removal (ROUT) method and excluded in all data. To analyze the association between different parameters, Spearman’s rank correlation coefficients were calculated. In figures, asterisks denote statistical significance (*, *p ≤ 0.05*; **, *p ≤ 0.01*; ***, *p ≤ 0.001*; ****, *p ≤ 0.0001*).

### Study approval

Animal experiments were approved by the local governmental commission for animal protection in Freiburg (Regierungspräsidium Freiburg, approval nos. G15/164, G18/80 and G21/98).

## Results

### Low vitamin D intake accelerates disease progression in lupus-prone NZB/W F1 mice

**Figure 1:**
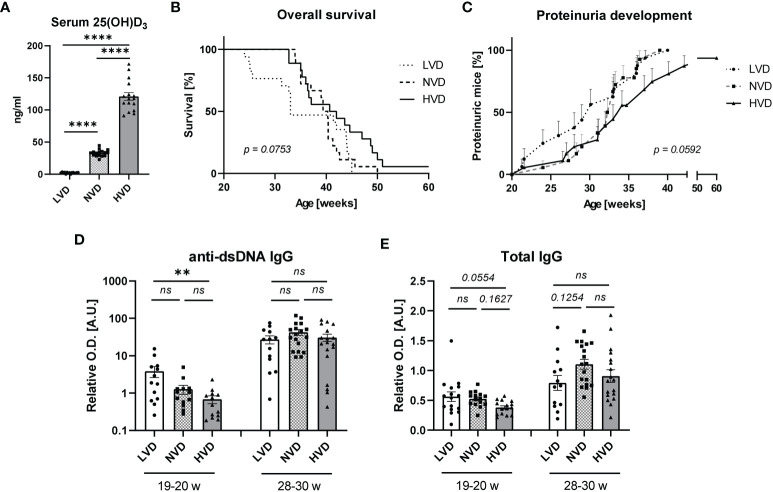
Low vitamin D (VD) intake, starting at 5 weeks of age, mildly accelerates disease progression in female lupus-prone NZB/W F1 mice. **(A)** Serum 25(OH)D_3_ concentrations were determined in 19–20-week-old female mice (n = 15–17), fed either a low-, normal-, or high-VD diet, with the latter containing 76,500 IU/kg. **(B)** Overall survival (OS) of LVD mice (n = 17), NVD mice (n = 18) and HVD mice (n = 18). The Kaplan–Meier method was used for estimating OS. Disease progression was monitored, including **(C)** proteinuria development, which was assessed semiquantitatively once a week, as well as serum **(D)** anti-dsDNA IgG and **(E)** total IgG titers, which were determined at an age of 19–20 weeks and 28–30 weeks in LVD mice (n = 13–15), NVD mice (n = 13–18), and (HVD mice (n = 13–18). Results are displayed as **(B)** survival curves, **(C)** time curves, and **(D, E)** scatter plots, with each data point representing an individual mouse in the latter. Data are expressed as **(C)** percents ± SEM or **(D, E)** mean ± SEM. *P ≤ 0.05* was considered significant, *p* ≥ *0.2* is indicated as *ns*, *not significant*. O.D., optical density; A.U., arbitrary unit; LVD, low vitamin D; NVD, normal vitamin D; HVD, high vitamin D. ** = p < 0.01; **** = p < 0.0001

VD deficiency is suggested to be an environmental risk factor for SLE, and the therapeutic potential of VD for SLE is being studied intensively. In light of this, we sought to explore the effects of long-term treatment with different dietary concentrations of cholecalciferol (VD) on lupus progression in female lupus-prone NZB/W F1 mice. Mice were continuously fed either a low-VD diet (<50 IU/kg; “LVD mice”), a normal-VD diet (500 IU/kg; “NVD mice”), or a high-VD diet (76,500 IU/kg; “HVD mice”) starting at 5 weeks of age. These dietary VD concentrations are based on previously published animal studies implementing comparable cholecalciferol concentrations (54–57), as well as human studies showing that high VD doses can be safely applied and may even be required to achieve VD sufficiency (58, 59). The measurement of serum concentrations of 25(OH)D_3_ (calcidiol), the main circulating VD metabolite, at 19–20 weeks of age revealed that the low-VD diet resulted in very low 25(OH)D_3_ concentrations (0–4 ng/ml; mean 2.2 ng/ml) indicative of severe VD deficiency (defined as <5 ng/ml in humans), while the normal-VD diet resulted in concentrations (22–45 ng/ml; mean: 32.9 ng/ml) that lie within the range of VD sufficiency for humans (30–60 ng/ml) and the high-VD diet produced high concentrations (90–171 ng/ml; mean: 121.2 ng/ml) in the upper range of tolerable VD levels and, in a few cases, in the lower end of the VD toxicity range for humans (>150 ng/ml) (60) (**Figure 1A**). Although no statistical significance was reached, our data point toward dose-dependent effects of VD on disease progression. LVD mice displayed the shortest OS and earliest onset of proteinuria, a clinical hallmark of LN (median survival: 33.0 weeks; median onset of proteinuria: 30.1 weeks), while NVD mice displayed intermediate OS and proteinuria onset (median survival: 39.9 weeks; median onset of proteinuria: 32.6 weeks) and HVD mice the longest OS and latest proteinuria onset (median survival: 41.4 weeks; median onset of proteinuria: 34.1 weeks) (**Figures 1B, C**). At 19–20 weeks of age, we detected significantly elevated serum anti-dsDNA IgG titers in LVD mice compared to HVD mice; in addition, there was a trend toward elevated total IgG concentrations in LVD mice. These differences were no longer observed at 28–30 weeks of age (**Figures 1D, E**).

**Figure 2:**
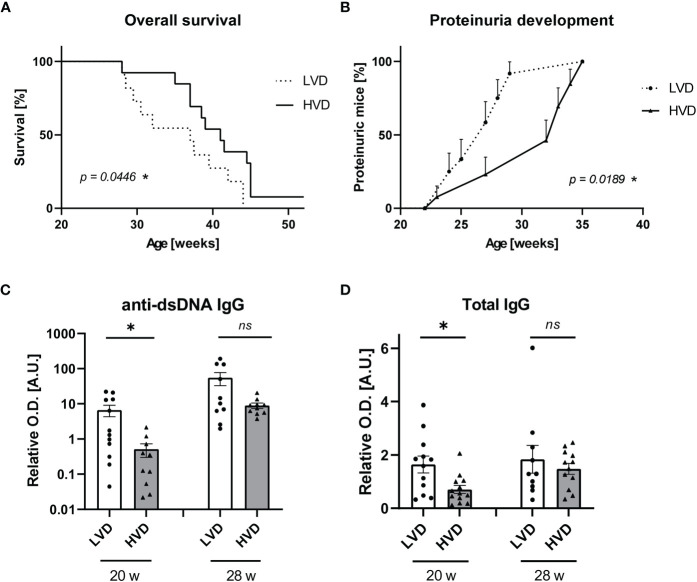
Low VD intake from the prenatal period onward accelerates disease progression in female lupus-prone NZB/W F1 mice. **(A)** OS of LVD mice (n = 11) and HVD mice (n = 13). The Kaplan–Meier method was used for estimating OS. Disease progression was monitored, including **(B)** proteinuria development, which was assessed semiquantitatively once a week, as well as serum **(C)** anti-dsDNA IgG and **(D)** total IgG titers, which were determined at an age of 20 and 28 weeks in LVD mice (n = 10–12), and HVD mice (n = 10–13). Results are displayed as **(A)** survival curves, **(B)** time curves, and **(C, D)** scatter plots, with each data point representing an individual mouse in the latter. Data are expressed as **(B)** percents ± SE or **(C, D)** mean ± SEM. *P ≤ 0.05* was considered significant, *p* ≥ *0.2* is indicated as *ns*, *not significant*. O.D., optical density; A.U., arbitrary unit; LVD, low vitamin D; HVD, high vitamin D. * = p < 0.05

The maternal VD status during pregnancy and early-life VD exposure have been suggested to be important determinants of immune regulation and the development of immune-mediated disorders in later life (61–68). When differential VD feeding commenced at 5 weeks of age, an age at which mice can almost be considered “adolescent” (69), we observed only mild effects of VD. Therefore, we decided to additionally explore the effects of VD when already administered during gestation and infancy and continued into adulthood. Since we observed the strongest differences between LVD and HVD animals, all further experiments were performed with a comparison of low-VD versus high-VD treatments only. In experiments involving maternal VD feeding, we reduced the cholecalciferol dose of the high-VD diet to 38,000 IU/kg, to avoid VD toxicity, which still produced comparably high serum concentrations of 25(OH)D_3_ in HVD mice (70–155 ng/ml; mean: 104.3 ng/ml) (**Supplementary Figure 1**). Following this approach, we did indeed observe stronger effects, with LVD mice exhibiting significantly reduced OS and significantly earlier proteinuria onset (**Figures 2A, B**). At 20 weeks of age, anti-dsDNA IgG and total IgG concentrations were once again significantly reduced in LVD mice (**Figures 2C, D**).

These differences in anti-dsDNA IgG were only significant at an early time point (20 weeks), when there was no relevant proteinuria detectable in the mice. At later time points, there was no difference in the concentrations of anti-dsDNA IgG and total IgG observed between the groups. This finding may indicate that beneficial effects of VD are more pronounced before the onset of disease or at the early stages of lupus. To obtain information on whether VD may also have prevented inflammation in the kidney, we performed immunofluorescence analysis for renal IgG deposits and determined *TLR*-7 and *TLR*-9 mRNA levels as a putative measure of downstream activation induced by the deposition of nucleic acid–containing immune complexes (ICs) in animals with beginning nephritis (25–26 weeks) (70, 71). We found that the concentrations of anti-dsDNA IgG in serum correlated with the amount of IgG deposits in kidneys, while no association was found between IC deposition or TLR-7/TLR-9 levels and proteinuria onset (**Supplementary Figures 3, 4**). Thus, it may be speculated that other, possibly VD-associated factors, than IC deposition influence the progression of nephritis and proteinuria.

To confirm that the reduced OS in LVD mice was not due to aberrant bone metabolism as a result of VD deficiency, bone density and grip strength were measured, revealing no significant differences between LVD and HVD mice (**Supplementary Figure 5**). Serum VD levels increased further upon longer exposure to the HVD diet, as shown when comparing the serum 25(OH)D_3_ levels of young (40–70 ng/ml; mean: 55.0 ng/ml) and older (70–155 ng/ml; mean: 104.3 ng/ml) HVD animals **Supplementary Figure 1**). These data suggest the accumulation of VD in the mice over time. However, HVD mice did not show signs of hypercalcemia due to VD intoxication, such as more frequent urination or increased water consumption, reduced/changed mobility indicative of fatigue or bone pain, weight loss, or reduced food intake indicative of reduced appetite or vomiting (data not shown).

Altogether, these results suggest detrimental effects of low VD intake on lupus progression in NZB/W F1 mice, with a stronger effect arising as a result of additional low maternal VD intake during gestation and lactation. The positive correlation between serum VD levels and age at proteinuria development as well as OS and the negative association with anti-dsDNA IgG support the dose-dependent beneficial effects of VD (**Supplementary Figure 2**). The fact that no such correlation was found within the individual groups indicates that rather global changes in the VD status matter, such as the presence of VD deficiency or not, while subtle changes within a high or low VD serum level range only have a minor impact (**Supplementary Table 2**).

### Low vitamin D intake reduces the differentiation of regulatory lymphocyte populations

In search of potential immunological effects of low VD intake that may contribute to the accelerated lupus progression in LVD mice, we compared the distribution, differentiation, and phenotype of adaptive and innate immune cell populations between LVD and HVD mice. This analysis was performed in the experiment where mice received either a low- or high-VD diet from the prenatal period onward, considering that under these conditions, effects on disease progression were most pronounced. We primarily explored these immunological parameters in 12–15-week-old male NZB/W F1 animals that do not display signs of lupus-like disease, since a skewed immune status, due to disease activity, is unlikely in these mice. This facilitates the identification of truly VD-mediated immunological effects. Considering that SLE patients have altered immune cell subset distribution, we additionally performed this immunological examination in 25–26-week-old female animals with established autoantibodies and beginning proteinuria, as these results might be of higher relevance for an active disease state.

**Figure 3:**
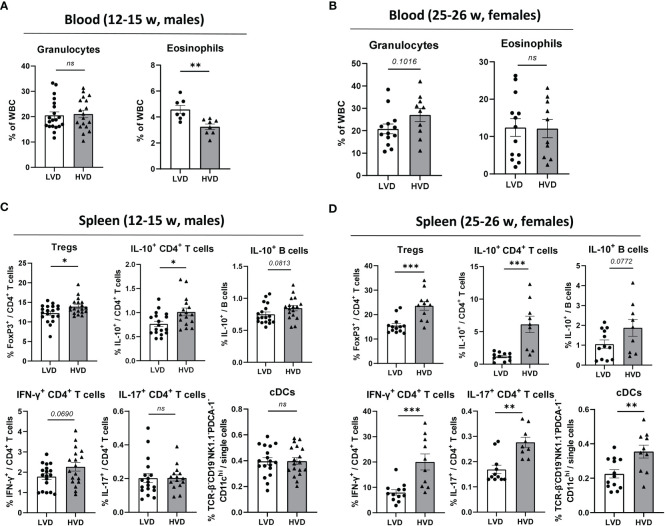
Low VD intake consistently hampers the adoption of a regulatory phenotype in lymphocytes. **(A, B)** Hematology analysis of granulocytes and eosinophils in the peripheral blood of **(A)** 12–15-week-old male and **(B)** 25–26-week-old female NZB/W F1 LVD and HVD mice. **(C, D)** Frequencies of Tregs, IL-10^+^ CD4^+^ T cells, IL-10^+^ B cells, IFN-γ^+^ CD4^+^ T cells, IL-17^+^ CD4^+^ T cells, and cDCs in the spleen of **(C)** 12–15-week-old LVD and HVD male NZB/W F1 mice, as well as **(D)** 25–26-week-old LVD and HVD female NZB/W F1 mice. Results are displayed as scatter plots, with each data point representing an individual mouse. Data are expressed as mean ± SEM. N/group: males = 7–20; females = 9–13. *P ≤ 0.05* was considered significant, *p* ≥ *0.2* is indicated as *ns*, *not significant*. Tregs, regulatory T cells; cDCs, conventional dendritic cells; LVD, low vitamin D; HVD, high vitamin D. * = p < 0.05; ** = p < 0.01; **** = p < 0.0001.

Upon examination of circulating blood cell populations, we found a significantly higher frequency of eosinophils in young healthy LVD mice, while total circulating granulocytes in these mice were unaffected (**Figure 3A**). However, this effect had vanished in 25–26-week-old female animals with beginning proteinuria (**Figure 3B**). While LVD females displayed elevated RBC counts and a higher frequency of circulating lymphocytes, in yet-healthy LVD males, RBCs, total WBCs, and other WBC subtypes were unchanged in the blood compared to HVD mice. (**Supplementary Figure 6**; **Supplementary Table 3**)

As previously described (53), the frequencies of B cells, plasma cells, CD4^+^ and CD8^+^ T cells, CD4^+^ T cell differentiation into Tregs and T_FH_ cells, IL-10 expression in B cells, and IL-10, IL-17A and IFN-γ expression in CD4^+^ and CD8^+^ T cells were explored in the spleen *via* flow cytometry. Within the compartment of innate immune cells, the frequencies of CD11b^+^ monocytic cells, conventional dendritic cells (cDCs), plasmacytoid dendritic cells (pDCs), NK cells and neutrophils were determined in the spleen (**Table 1**). The only consistent effects we could identify in both healthy male and in early-diseased female mice were the significantly decreased frequencies of Tregs and IL-10^+^ CD4^+^ T cells in LVD mice as well as a trend of lower frequencies of IL-10^+^ B cells, suggesting that low VD intake reduces the differentiation of these lymphocyte populations (**Figures 3C, D**). Low VD intake did not result in any shifts in the distribution of main innate immune cell populations in the spleen of male animals, and only LVD females displayed decreased frequencies of cDCs (**Table 1**; **Supplementary Figure 7**; **Figure 3D**). The frequencies of other effector T cell subsets, including IL-17^+^ CD4^+^ T cells, T_FH_ cells, and IFN-γ^+^ CD4^+^ and CD8^+^ T cells, were unchanged between LVD and HVD males, which was also true for B-cell subsets such as germinal center (GC) B cells and plasma cells. Among females, however, HVD mice displayed significantly increased frequencies of IL-17^+^ CD4^+^ T cells, as well as IFN-γ^+^ CD4^+^, and CD8^+^ T cells (**Table 1**; **Figure 3D**).

**Table 1:** Immunological changes in spleen of LVD and HVD mice.

	12-15 w (males)			25-26 w (females)		
Spleen	LVD	HVD		LVD	HVD	
	Mean +/- SEM	Mean +/- SEM	*P-value*	Mean +/- SEM	Mean +/- SEM	*P-value*
**Adaptive immune cells**						
**Distribution of immune cell populations**						
**CD4^+^ T cells**: TCR-β^+^B220^-^CD4^+^CD8^-^ / single cells [%]	22.12 +/- 0.43	20.80 +/- 0.61	*0.0910*	22.83 +/- 0.86	22.33 +/- 0.65	*ns*
**Tregs**: FoxP3^+^ / CD4^+^ T cells [%]	12.25 +/- 0.50	13.92 +/- 0.45	0.0219*	15.50 +/- 0.98	23.62 +/- 1.54	0.0003***
**T_FH_ cells**: CXCR5^hi^PD-1^hi^ / CD4^+^ T cells [%]	0.450 +/- 0.057	0.588 +/- 0.086	*ns*	1.132 +/- 0,212	2.167 +/- 0,406	*0.0666*
**CD8^+^ T cells**: TCR-β^+^B220^-^CD8^+^CD4^-^ / single cells [%]	16.49 +/- 0.32	15.96 +/- 0.56	*ns*	15.49 +/- 0.81	12.75 +/- 1.40	*0.1168*
**B cells**: TCR-β^-^B220^+^ / single cells [%]	37.82 +/- 0.63	39.42 +/- 0.72	*0.1277*	35.84 +/- 2.23	36.93 +/- 2.02	*ns*
**GC B cells**: Fas^hi^GL7^hi^ / B cells [%]	0.525 +/- 0.079	0.542 +/- 0.064	*ns*	1.302 +/- 0.332	2.284 +/- 0.529	*0.1306*
**Plasma cells**: CD138^hi^ / single cells [%]	0.497 +/- 0.037	0.543 +/- 0.032	*ns*	1.492 +/-0.086	1.643 +/- 0.137	*ns*
**Co-stimulatory molecules**						
B cells: CD86 MFI CD80 MFI ICOS-L MFI	557.4 +/- 10.17 121.8 +/- 7,03 469.4 +/- 30.72	558.6 +/- 9.77 134.3 +/- 6.58 484.8 +/- 44.67	*ns* *0.1039* *ns*			
**Cytokines**						
CD4^+^ T cells:						
IFN-γ^+^ / CD4^+^ T cells [%]	1,787 +/- 0.139	2.265 +/- 0.205	*0.0690*	8.247 +/- 1.252	20.030 +/- 1.335	0.0006***
IL-17^+^ / CD4^+^ T cells [%]	0.202 +/- 0.026	0.204 +/- 0.019	*ns*	0.169 +/- 0.006	0.277 +/- 0.010	0.0017**
IL-10^+^ / CD4^+^ T cells [%]	0.766 +/- 0.052	1.014 +/- 0.077	0.0132*	3.136 +/- 0.410	10.608 +/- 1.043	0.0018**
CD8^+^ T cells						
IFN-γ^+^ / CD8^+^ T cells [%] B cells IL-10^+^ / B cells [%]	2.707 +/- 0.147 0.751 +/- 0.035	2.519 +/- 0.147 0.847 +/- 0.041	*ns* *0.0813*	3.995 +/- 1.201 2.417 +/- 0.202	12.383 +/- 2.650 3.613 +/- 0.368	0.0002*** *0.1158*
**Innate immune cells**						
**Distribution of immune cell populations**						
**cDCs**: TCR-β^-^CD19^-^NK1.1^-^PDCA-1^-^CD11c^hi^ / single cells [%]	0.396 +/- 0.026	0.394 +/- 0.019	*ns*	0.226 +/- 0.024	0.355 +/- 0.032	0.0066**
**pDCs**: TCR-β^-^CD19^-^NK1.1^-^PDCA-1^hi^ / single cells [%] **NK cells:** TCR-β^-^CD19^-^NK1.1^+^ / single cells [%]	0.646 +/- 0.078 6.116 +/- 0.173	0.663 +/- 0.076 6.329 +/- 0.188	*ns* *ns*	0.351 +/- 0.033 4.672 +/- 0.178	0.371 +/- 0.028 5.434 +/- 0.308	*ns* *0.0506*
**Neutrophils:** TCR-β^-^CD19^-^NK1.1^-^CD11b^+^Ly6G^hi^/ single cells [%]	0.128 +/- 0.025	0.148 +/- 0.025	*ns*	0.391 +/- 0.072	0.374 +/- 0.058	*ns*
**CD11b^+^ Monocytic cells**: TCR-β^-^CD19^-^NK1.1^-^CD11b^+^Ly6G^-^ / single cells [%]	0.600 +/- 0.087	0.558 +/- 0.068	*ns*	0.245 +/- 0.034	0.352 +/- 0.047	*0.0946*
**Ly6C^hi^ CD11b^+^ Monocytic cells**: Ly6C^hi^ / CD11b^+^ Monocytic cells [%]	61.97 +/- 2.59	61.14 +/- 2.27	*ns*	52.08 +/- 3.89	47.88 +/- 3.12	*ns*
**Co-stimulatory molecules**						
cDCs:						
CD86 MFI	994.7 +/- 29.56	982.9 +/- 22.21	*ns*			
CD80 MFI	1056.9 +/- 46.87	1035.4 +/- 48.32	*ns*			
ICOS-L MFI	573.6 +/- 27.45	597.7 +/- 30.14	*ns*			
Total CD11b^+^ Monocytic cells:						
CD86 MFI	875.9 +/- 38.16	800.2 +/- 31.83	*0.1453*			
CD80 MFI	239.1 +/- 7.30	235.6 +/- 10.4	*ns*			
ICOS-L MFI	1137.9 +/- 66.56	1138.6 +/- 53.50	*ns*			

Broad immune status evaluation in the spleen of 12-15-week-old LVD and HVD male, as well as 25-26-week-old LVD and HVD female NZB/W F1 mice via flow cytometry. Results are expressed as mean +/- SEM. N/group: males = 7-20; females = 9-13. P ≤ 0.05 was considered significant, p ≥ 0.2 is indicated as ns, not significant. Asterisks denote statistical significance (*, p ≤ 0.05; **, p ≤ 0.01; ***, p ≤ 0.001). LVD, low vitamin D; HVD, high vitamin D.

To summarize, these data reveal the consistent inhibitory effects of low VD intake on the differentiation of regulatory lymphocyte populations and sporadic somewhat pro-inflammatory effects in early-diseased animals; the latter might have been provoked by the long-term intake of high VD doses or the inflammatory milieu.

### Low vitamin D intake has no consistent effects on phenotype and function of innate immune cells

Innate immune cells are reported to play a significant role in lupus pathogenesis, with their phagocytic capacity and cytokine production having been shown to be abnormal in SLE patients (4–10). An increased production of pro-inflammatory cytokines is also characteristic of innate immune cell reprogramming, termed “trained immunity,” induced by not only infectious but also sterile triggers such as Western dietary habits (72–76).

**Figure 4:**
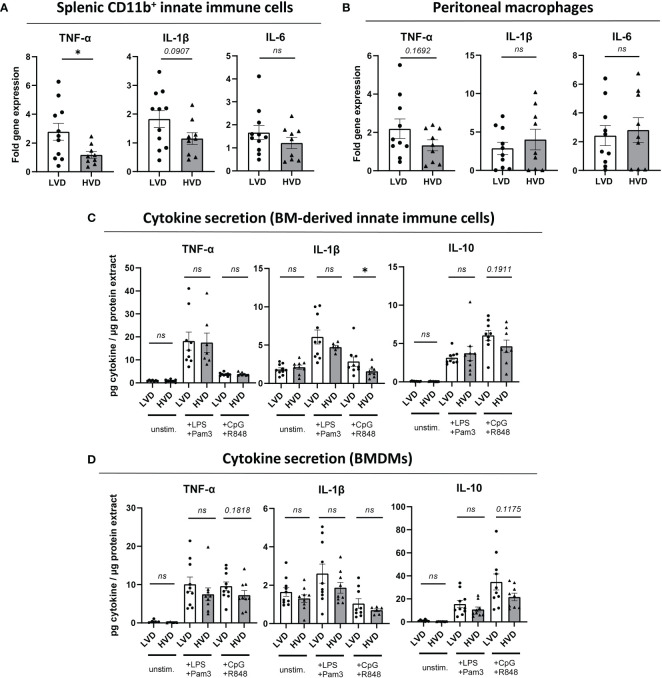
Low VD intake has no consistent effects on cytokine production in innate immune cells. **(A, B)** Fold gene expression of pro-inflammatory cytokines, following stimulation with 50 ng/ml LPS for 2 h 45 min, in **(A)** splenic CD11b^+^ innate immune cells and **(B)** peritoneal macrophages of 12–15-week-old male NZB/W F1 mice. **(C, D)** Cytokine levels in culture supernatants of **(C)** BM-derived innate immune cells (+ GM-CSF) and **(D)** BMDMs (+ M-CSF) from 12–15-week-old male NZB/W F1 mice, following stimulation with either 50 ng/ml LPS and 50 ng/ml Pam3CSK4, or 1 µM CpG and 1 µM R848, for 20 h, normalized to total protein content of adherent cells. Results are displayed as scatter plots, with each data point representing an individual mouse. Data are expressed as mean ± SEM. N/group: LVD = 9-11; HVD = 6-9. *P ≤ 0.05* was considered significant, *p* ≥ *0.2* is indicated as *ns*, *not significant*. BM, bone marrow; BMDMs, BM-derived macrophages; Pam3, Pam3CSK4; unstim., unstimulated; LVD, low vitamin D; HVD, high vitamin D. * = p < 0.05

Having observed no differences in the distribution of main innate immune cells between LVD and HVD mice (**Table 1**; **Supplementary Figure 7**), we examined in more detail the impact of low VD intake on their functionality and the potential adoption of a trained immunity phenotype. To that end, as reported by Christ et al. (76), we investigated whether our dietary modification would affect the expression of costimulatory molecules and cytokine production in innate immune cells from different sites in young male mice. The production of the pro-inflammatory cytokines TNF-α, IL-1ß, and IL-6 in purified splenic CD11b^+^ innate immune cells and peritoneal macrophages was explored by quantitative reverse transcription PCR, after short-term stimulation with LPS (**Supplementary Figure 13**). In addition, we explored cytokine production in BM cells after 5-day differentiation into BM-derived innate immune cells, comprising a mix of dendritic cells and macrophages, and BM-derived macrophages (BMDMs), *via* the addition of GM-CSF or M-CSF, respectively (77, 78). Here, the secretion of TNF-α, IL-1ß, and IL-10 was determined by ELISA after 20 h stimulation with a panel of TLR ligands. Purified splenic CD11b^+^ innate immune cells displayed significantly increased *TNF* mRNA levels and a trend of elevated *IL1B* mRNA levels in LVD mice **(** **Figure 4A** **)**, while this effect was not reflected in purified peritoneal macrophages (**Figure 4B**). In contrast, we found no significant differences in cytokine secretion by BM-derived innate immune cells or BMDMs between LVD and HVD mice (**Figures 4C, D**).

Trained immunity has also been reported to entail an increased expression of costimulatory molecules (75, 76, 79, 80). Thus, we measured the surface expression of CD86, CD80, and ICOS-L on splenic cDCs and CD11b^+^ monocytic cells, circulating CD11b^+^ monocytic cells and peritoneal macrophages. We observed no differences between LVD and HVD mice (**Supplementary Figure 9**). In support of this, we also found no increase in the frequencies of splenic or circulating Ly6C^hi^CD11b^+^ monocytic cells, which are considered pro-inflammatory and have been reported to be increased following the induction of trained immunity (**Supplementary Figure 10A**) (81–84).

**Figure 5:**
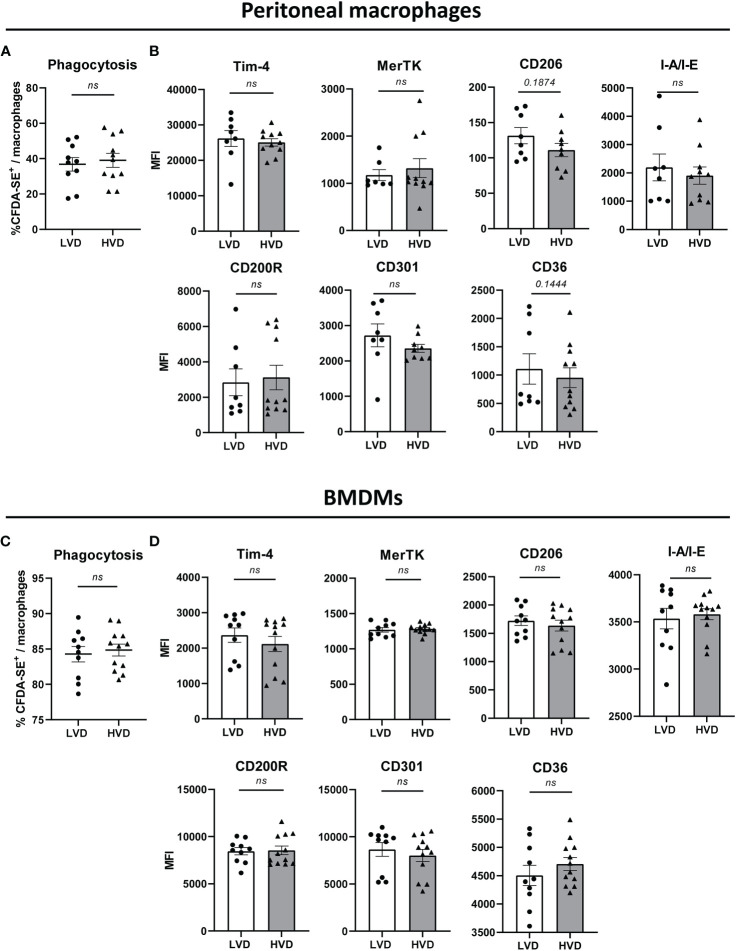
Low VD intake has no effect on phagocytic capacity and surface expression of phagocytosis-associated markers in macrophages. **(A, C)** Phagocytic uptake of apoptotic thymocytes by **(A)** peritoneal macrophages and **(C)** BM-derived macrophages (BMDMs) of 12–15-week-old LVD (n = 10) and HVD (n = 11-12) male animals. **(B, D)** Surface expression of phagocytosis-associated markers by **(B)** peritoneal macrophages and **(D)** BMDMs of 12–15-week-old LVD (n = 7-10) and HVD (n= 10-12) male animals. Results are displayed as scatter plots, with each data point representing an individual mouse. Data are expressed as mean ± SEM. *P ≤ 0.05* was considered significant, *p* ≥ *0.2* is indicated as *ns*, *not significant*. MFI, mean fluorescence intensity; BMDMs, BM-derived macrophages; LVD, low vitamin D; HVD, high vitamin D.

Impaired phagocytosis of apoptotic cells by macrophages has been implicated in SLE pathogenesis. Therefore, we next determined the effect of low VD intake on the phagocytosis of apoptotic cells by both peritoneal macrophages and BMDMs, the latter being generated by a 5-day culture of BM cells with M-CSF. Phagocytosis was measured by co-culturing CD45.2^+^ macrophages with CD45.1^+^ CFDA-SE-labeled apoptotic thymocytes (ATs) and quantifying phagocytes that had phagocytosed ATs as CD45.2^+^CD45.1^-^CD11b^+^F4/80^+^CFDA-SE^+^ cells *via* flow cytometry (**Supplementary Figure 14**). As CD45.2^+^CD45.1^+^CFDA-SE^+^ cells represent cells with non-ingested ATs attached to their surface, these cells were not counted. In addition to measuring phagocytic capacity, we examined the expression of surface markers associated with phagocytosis. Between LVD and HVD mice, there were no differences in the phagocytic capacity of peritoneal macrophages or BMDMs, or the surface expression of phagocytosis-associated markers on these cells (**Figure 5**).

Together, these data do not suggest stringent effects of low VD intake on the phenotype and function of innate immune cells, including cytokine production, costimulatory molecule expression, and phagocytic capacity.

## Discussion

Environmental factors are becoming increasingly acknowledged as triggers of the development of autoimmune diseases such as SLE. Since VD is known to exert immunomodulatory effects and there is a high prevalence of VD deficiency among SLE patients, VD deficiency has been thoroughly contemplated as a risk factor for SLE (21–27). In this study, we explored the effects of dietary VD on lupus-prone NZB/W F1 mice and attempted to identify the immunological effects of low VD intake that could promote lupus progression. We report that low VD intake accelerates lupus progression, with this effect becoming significant when low VD intake commenced during the prenatal period. Our data reveal that low VD intake reduces the differentiation of immunoregulatory lymphocyte populations. Thus, these data add to the understanding of how VD deficiency may play a role in promoting lupus development.

While many studies demonstrate an inverse relationship between VD levels and disease activity in SLE patients (85–87), the collective data from interventional studies investigating the effects of oral VD supplementation on SLE disease activity are inconsistent (88–91). Contradictory results may, in part, be due to variations in the administered cholecalciferol dose, study design, and placebo group, as well as different treatment durations. The age groups of cohorts also differ between studies, since some studies investigate the effect of oral VD supplementation in pediatric or juvenile patients, while most others involve adults. Age-dependent effects were also addressed in our study. Our data demonstrating the detrimental effects of low VD feeding on lupus progression did not achieve statistical significance when fed from an age of 5 weeks onward, in contrast to when low VD intake commenced during the prenatal period. This suggests that the maternal VD status during gestation may play a role in autoimmune development in offspring. Some, but not all studies, support the notion of a role of the maternal VD status during pregnancy in the risk of the development of immune-mediated diseases in offspring (61–66, 92–96). VD is known to be a potent epigenetic modulator, and epigenetic abnormalities have been reported in SLE (32, 33, 97, 98). Interestingly, both human and mouse studies have demonstrated that the maternal VD status during pregnancy affects DNA methylation in offspring, including genes involved in apoptosis regulation and immune function (99, 100). Furthermore, it has been suggested that the effectiveness of an individual’s response to VD can vary and that individuals can be categorized as high-, mid-, or low-VD responders (101, 102). Accordingly, there is some dispute on the desired VD status and the recommended daily intake. Our results support the recommendations of sufficient daily intake of VD and the relevance of avoiding VD deficiency. However, and as discussed below, our data and others also show that it needs to be considered that very high VD levels over a longer period may not only bear the risk of hypercalcemia but also, even below toxic levels, they might induce somewhat pro-inflammatory changes with potentially adverse effects on the progression of autoimmune pathology (54, 103). As this was not reported by other trials (58, 59), more work is required to elucidate the relevance and impact of VD dosage in health and disease.

To the best of our knowledge, there have been no studies investigating the effect of dietary cholecalciferol feeding on murine lupus pathology. As an alternative to oral cholecalciferol treatment, many preclinical studies employ the application of calcitriol, the active VD metabolite. Calcitriol application may generally induce stronger effects than oral cholecalciferol supplementation, since direct calcitriol application bypasses regulatory steps within physiological VD metabolism (104, 105). Two studies involving lupus-prone MRL/l mice receiving calcitriol treatment show effectiveness in ameliorating disease (106, 107). We also explored the effects of continuous calcitriol injections in female NZB/W F1 mice from 5 weeks of age onward and found that it neither improved OS and proteinuria, nor inhibited autoantibody production (data not shown). As calcitriol application resulted in slight weight loss and a mild deterioration of general health in some animals, interfering side effects by the continuous application were likely. Therefore, a long-term treatment of mice with calcitriol may entail difficulties, in contrast to cholecalciferol, which has a wider therapeutic index.

We observed that low VD intake resulted in elevated anti-dsDNA IgG and total IgG concentrations. This finding is in concordance with a study by Terrier et al. that reported a significant reduction in anti-dsDNA levels in VD-deficient SLE patients, following an oral cholecalciferol supplementation regimen (108). The fact that we observed no significant difference in autoantibody titers between the groups at 28 weeks of age may suggest that VD primarily delays the rise in autoantibody titers during early disease pathogenesis (20 weeks) and that later during pathogenesis (28 weeks), the moderate effects of VD are no longer sufficient to dampen the production of autoantibodies. Indeed, it has been observed in the context of a different experimental model that the immunoregulatory effects of VD are overridden by strong immune activity (109). As discussed below, it could also be speculated that increasingly high VD levels might, over time, weaken the initially preponderant immunoregulatory effects. Alternatively, increased proteinuria in 28-week-old LVD mice may result in the increased renal loss of immunoglobulins, thereby resulting in the decreased serum concentrations of anti-dsDNA as well as total IgG.

Alongside autoantibody-mediated IC deposition, there are other factors that contribute to the development of nephritis and proteinuria that may be regulated by VD. Indeed, VD and VD analogs have various antiproteinuric and anti-inflammatory effects on the kidneys (110–115). Thus, it is possible that the accelerated proteinuria development we observed in LVD mice was due to a combination of elevated autoantibody titers and a lack of VD-mediated nephroprotective effects. Some data exist on the effects of VD in the context of LN; a recent study put the nephroprotective effects of VD to the test and reported that *in vitro* calcitriol treatment protects podocytes from autoantibody-induced injury by reducing aberrant autophagy (116). Moreover, the treatment of MRL/l mice with a calcitriol analog inhibits the development of LN (117). Mechanistically, VD was reported to exert its antiproteinuric effect by inhibiting the expression of heparanase and by modulating the renin–angiotensin–aldosterone system (110, 111). In addition, immunological effects of VD and VD analogs have been reported in mouse models of renal fibrosis and obstructive nephropathy, such as a reduction of IL-6 and IL-1β in the kidney and reduced T cell and macrophage infiltration (113, 115). We also examined the immunological effects of low VD intake, starting at 5 weeks of age, in the kidneys of 27-week-old female animals with beginning nephritis, but observed no significant differences between LVD and HVD mice in the frequencies of immune cells (data not shown). In contrast to our data and numerous studies demonstrating the nephroprotective effects of VD, Vaisberg et al. reported a worsening of histopathological findings in the kidneys of female NZB/W F1 mice receiving long-term intraperitoneal (i.p.) cholecalciferol treatment (118). The possible explanations for these contradictory results remain elusive. We can only speculate that application route or dose might play a role.

Generally, our results suggest that VD could have beneficial effects on the early disease phase and delay its onset, while during a later stage, it may limit nephritis progression. The beneficial impact on the early disease phase is supported by reduced anti-dsDNA IgG in 20-week-old HVD female mice and the increased frequencies of regulatory lymphocyte subsets in yet-healthy HVD animals. Further, our data do not prove, but indirectly suggest, that other factors than IC deposition might influence the progression of nephritis and proteinuria, such as renal VD-mediated effects, since we found a correlation between anti-dsDNA IgG in serum and IgG deposition in the kidney but no association between IgG deposition or renal TLR-7/TLR-9 levels and proteinuria onset. However, some limitations need to be considered; immunofluorescence only allows a semiquantitative analysis of IC deposition. Moreover, the disease activity of LN may be influenced by other factors than IC deposition, such as IC composition, autoantibody subclass as well as glycosylation pattern, renal cellular composition, and cellular activation and differentiation status. The exact significance of the causative relationship between IC quantity versus other factors and nephritis progression is not clearly established. As these indications are only indirect, future studies should address in more detail the important question at which phase of disease pathogenesis exactly VD exerts its beneficial effects.

Given the effects of low VD intake on autoantibody production and lupus progression, we suspected immunological effects. We observed an increase in the frequency of circulating eosinophils in yet-healthy LVD mice, while the total circulating granulocytes in these mice were not elevated, suggesting increased eosinophil differentiation. This is supported by human studies demonstrating a negative correlation between serum VD and blood eosinophil levels (119–121). However, in SLE, the role of eosinophils has hardly been studied. While there are a small number of case studies of patients with SLE and various other autoimmune disorders displaying hypereosinophilic syndrome (HES) (122–126), it is not evident whether HES precedes SLE or vice versa. Similarly, the role of eosinophils in LN is not well explored. One study reported an increased frequency of eosinophiluria in LN patients compared to SLE patients without renal involvement (127). However, this does not necessarily implicate eosinophils in LN pathogenesis. On the other hand, in another study on patients with various immune and non-immune kidney diseases, peripheral eosinophilia was associated with increased renal eosinophil infiltration and progression to end-stage kidney disease (128). Since we no longer observed elevated levels of circulating eosinophils in females with beginning nephritis, it is possible that this could indeed be due to increased renal eosinophil infiltration, although this needs to be investigated. As eosinophils are potent producers of the plasma cell survival factor APRIL (129, 130), one could further speculate about them representing an important factor in sustaining lupus and nephritis progression.

As the most consistent immunological effect occurring in both healthy animals and those with beginning disease, we observed an inhibitory effect of low VD intake on the differentiation of regulatory lymphocyte populations, encompassing Tregs and IL-10-producing lymphocytes. This is in concordance with previous studies showing that VD promotes Treg differentiation and augments IL-10 production in CD4^+^ T cells and B cells (42–44, 51, 57, 131). This is relevant in the context of lupus since Tregs and IL-10 play an established and important immunoregulatory role in constraining the development of autoimmunity (132–139). Furthermore, IL-10-producing putative regulatory B cells have also been reported to exert protective effects in lupus-prone mice (140, 141). However, IL-10 possesses context-dependent dual functions that can also involve pro-inflammatory effects and may support antibody responses (142–144). Only in female mice with beginning disease did we note sporadic pro-inflammatory immunological effects in the HVD group, including the increased frequencies of cDCs, IL17^+^ and IFN-γ^+^ T cells. Considering that most studies have reported the ability of VD to lower the expression of pro-inflammatory cytokines, such as IFN-γ (145–147), these results were surprising at first. On the other hand, some studies report enhancing effects of VD on IFN-γ expression (148, 149). In our study, the significance and cause of the sporadic pro-inflammatory changes observed in diseased HVD mice remain unresolved. A possible explanation might be that inflammation and the disease context could have modulated the effects of VD on immune cells. In addition, the adverse effects of very high and long-term serum 25(OH)D_3_ concentrations in the upper tolerable range cannot be excluded. In support of such a scenario, Häusler et al. have shown that mice supplemented with high doses of cholecalciferol and displaying high serum VD levels developed fulminant experimental autoimmune encephalomyelitis (EAE) with a massive infiltration of the central nervous system by activated myeloid cells, T_H_1 and T_H_17 cells. In contrast, moderate supplementation reduced EAE severity along with an expansion of Tregs (54). Considering that VD seemed to successively accumulate in our mice fed the high-VD diet, there may be similar effects explaining our observations; in young HVD mice displaying serum VD levels in a healthy range, only an increase in putatively beneficial immunoregulatory lymphocyte subsets was found, while in older HVD mice with markedly elevated serum VD levels, additional and putatively pro-inflammatory immune effects comparable to those reported by Häusler et al. (54) were noted. As high VD intake still conferred beneficial effects on disease pathology in our study, we suspect that beneficial high VD effects may have outweighed pro-inflammatory effects occurring at later disease stages. This may also explain why serum anti-dsDNA IgG was only decreased in HVD mice at 20 but not 28 weeks of age. Further, and as discussed above, VD may exert additional nephroprotective effects limiting nephritis progression. This underlines the importance of exploring the influence of VD dosage, as still, it is not fully resolved whether normal or rather high-normal serum VD levels should be aimed for to reach optimal beneficial effects in inflammatory immune-mediated diseases and autoimmunity (103, 150–152). To address these questions, future studies should monitor more closely the development of disease and immunological changes in relation to serum VD levels and the intake of different VD doses. Thus, in retrospect, one limitation of our study is that immunological effects have not additionally been explored for the NVD group.

We also explored the effects of VD on the phenotype and function of innate immune cells in more detail. Dysregulated production of pro-inflammatory cytokines plays a central role in autoimmunity. An increased production of pro-inflammatory cytokines is characteristic of a trained immunity phenotype in innate immune cells, and it has been argued that monocytes and macrophages from patients with autoimmune disease display such features (8, 9, 72). Alongside infectious triggers, environmental factors can also induce such reprogramming of innate immune cells, as previously shown by a study on myeloid cells from mice fed a Western diet (76). We therefore sought to investigate whether low VD intake similarly affects cytokine production, since VD regulates the epigenome and epigenetic rewiring underlies the adoption of a trained immunity phenotype (153, 154). We examined cytokine production by various innate immune cells but only observed increased pro-inflammatory cytokine production in splenic CD11b^+^ innate immune cells of LVD mice. In accordance with the overall inconsistent effects of VD on the cytokine milieu, we did not observe any differences between LVD and HVD mice in the upregulation of costimulatory molecules in innate immune cells, which is another feature of trained immunity (75, 76, 79, 80). In summary, our data demonstrate the effects of VD on the cells of the adaptive immune system but do not suggest a major impact on the cytokine production, phagocytic capacity, or activation status of innate immune cells in the employed setting.

In conclusion, low VD intake has detrimental effects on the manifestation and progression of lupus-like disease in NZB/W F1 mice. Since SLE is a multifactorial disease with an important contribution of environmental factors to disease manifestation, it is likely that VD deficiency represents an important environmental factor that may tip the balance toward the development of manifest autoimmunity. Therefore, adequate VD intake remains important in SLE, especially for VD-deficient patients. Moreover, sufficient VD intake may delay or prevent the manifestations of SLE in individuals at risk, such as relatives of SLE patients, especially if they are positive for antinuclear antibodies. Further research is necessary to better understand the immunological mechanisms underlying the effects of VD in lupus and to determine optimal VD dosage and serum concentrations.

## Data availability statement

The original contributions presented in the study are included in the article/**Supplementary Material**. Further inquiries can be directed to the corresponding authors.

## Ethics statement

The animal study was reviewed and approved by local governmental commission for animal protection of Freiburg (Regierungspräsidium Freiburg, approval nos. G15/164, G18/80 and G21/98).

## Author contributions

NC, REV, ANK, A-LS, and DTLS designed research. NC, ANK, A-LS, DTLS, JPW, LR, QA, AL, ZW, and KH performed research. NC, ANK, A-LS, DTLS, J-DF, MHe, and BS analyzed data. HD and MHo contributed new reagents/analytical tools. NC, ANK, A-LS, DTLS, BS, JPW, LR, QA-K, HD, KH, MHo, MHe, GH, and REV contributed to manuscript editing. ANK and NC wrote the paper. All authors contributed to the article and approved the submitted version.

## Funding

This work was supported by the Ministry of Science, Research, and Arts Baden-Wurttemberg (Margarete von Wrangell Programm to NC and MHo), Deutsche Forschungsgemeinschaft (DFG) (TRR 130, project 12 to RV and NC and project 19 to GH; SFB 1160 project 12 to RV and project A02 to MHo), and the Erika Bürgy Fundation (Stiftung für die Region – Sparkasse Pforzheim Calw Treuhandstiftung Erika Bürgy Stiftung) to RV, as well as the Medical Faculty of the University of Freiburg (MOTI-VATE fellowship to JW and AL). This project was cofinanced by the European Regional Development Fund (ERDF) of the European Union in the framework of the INTERREG V Upper Rhine programme (project PERSONALIS).

## Acknowledgments

We thank all staff of the animal facility (CEMT) for help with animal care and husbandry and Rita Rzepka and Ingeborg Wünsche for organizational and infrastructural measures. The bone density measurement was performed in the Institute of Radiology, Preclinical Imaging Platform Erlangen (PIPE), Friedrich-Alexander Universität Erlangen and was supported by DFG SFB1181 project Z02 to Tobias Bäuerle.

## Conflict of interest

The authors declare that the research was conducted in the absence of any commercial or financial relationships that could be construed as a potential conflict of interest.

## Publisher’s note

All claims expressed in this article are solely those of the authors and do not necessarily represent those of their affiliated organizations, or those of the publisher, the editors and the reviewers. Any product that may be evaluated in this article, or claim that may be made by its manufacturer, is not guaranteed or endorsed by the publisher.
