# Supplementary material for: Impact of dietary vitamin D on immunoregulation and disease pathology in lupus-prone NZB/W F1 mice
Source: Front Immunol. 2022 Nov 24;13:933191. doi: 10.3389/fimmu.2022.933191 (PMC9730823; doi:10.3389/fimmu.2022.933191)
Supplement: Supplementary file 1 [file DataSheet_1.pdf]

Supplementary Material

Supplementary Figures

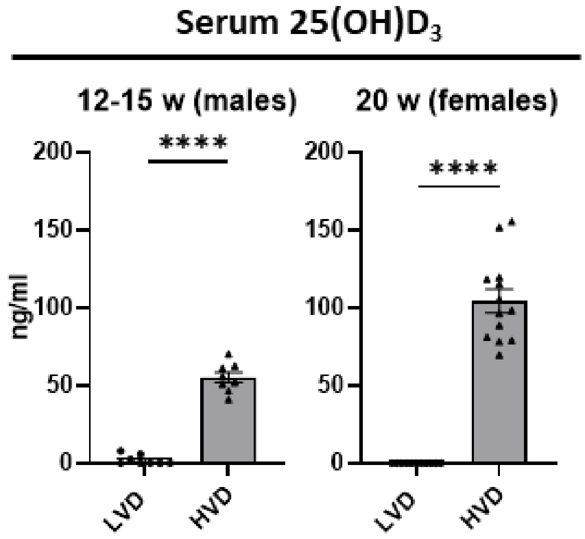

Supplementary Figure 1: Impact of the low vitamin D and high vitamin D diets on serum 25(OH)D_3 concentrations in NZB/W F1 mice. Serum 25(OH)D_3 concentrations were determined in 12-15-week-old male (n = 8) and 20-week-old female (n = 11-13) mice, fed either a low or high VD diet, with the latter containing 38,000 IU/kg. Results are depicted as scatter plots, with each data point representing an individual mouse. Data are expressed as mean ± SEM. $P \leq 0.05$ was considered significant, $p \geq 0.2$ is indicated as *ns*, *not significant*. Abbreviations: VD = vitamin D; LVD = low vitamin D; HVD = high vitamin D.

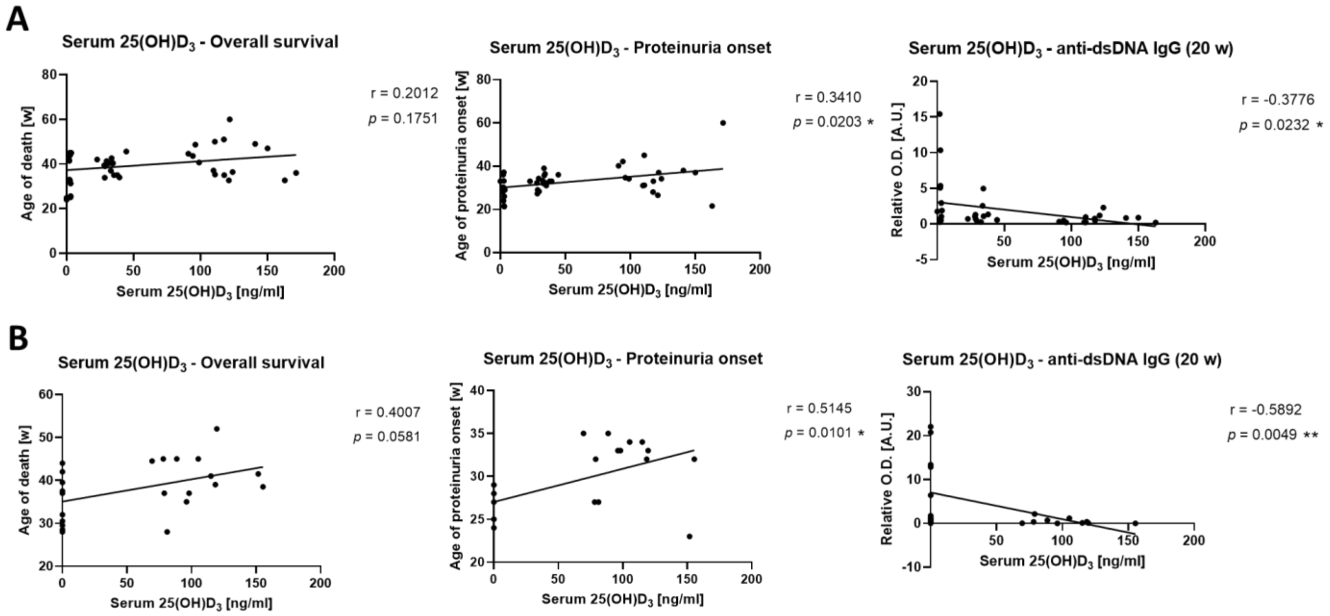

Supplementary Figure 2: Association between serum VD levels and survival, proteinuria onset and anti-dsDNA IgG. (A, B) The relationship between serum 25(OH)D_3 concentrations and overall survival, proteinuria onset and serum anti-dsDNA IgG titers was analyzed by Spearman's rank correlation. This was determined in **(A)** female mice fed either a low, normal or high vitamin D (VD) diet, with the latter containing 76,500 IU/kg, as well as **(B)** female mice fed either a low or high VD diet, with the latter containing 38,000 IU/kg. Graphed are numerical values, Spearman's rank correlation coefficient and its strength. $P \leq 0.05$ was considered significant, $p \geq 0.2$ is indicated as *ns*, *not significant*. Abbreviations: VD = vitamin D.

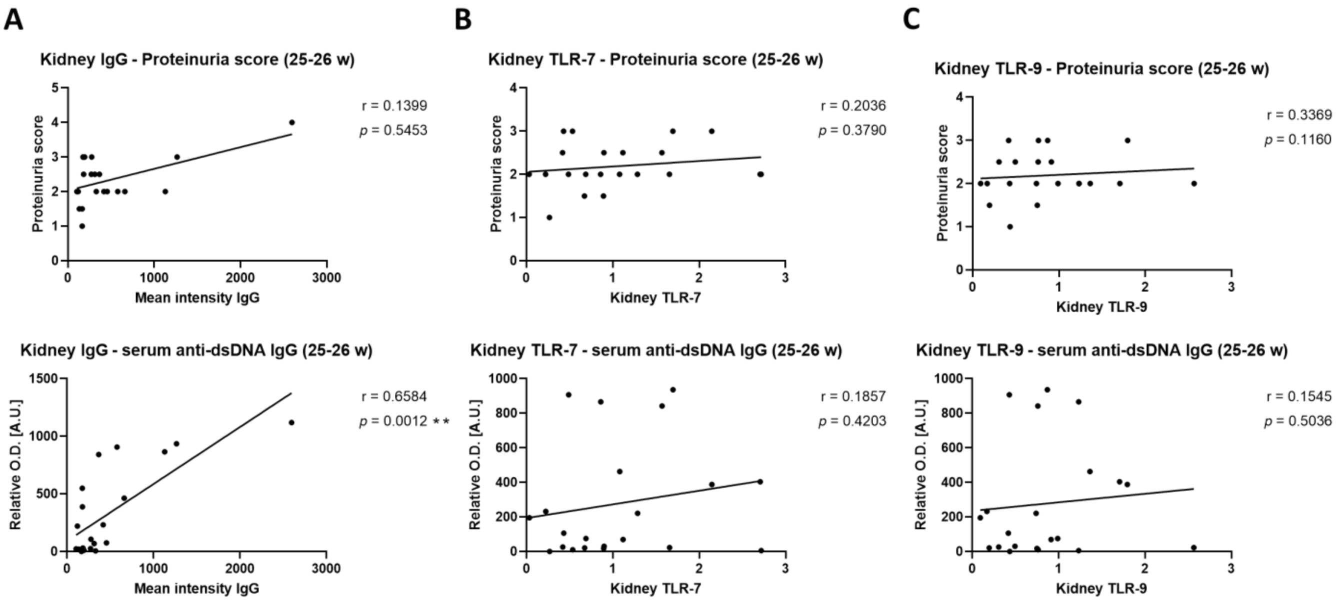

Supplementary Figure 3: Association between IgG deposits in glomeruli, as well as renal expression of TLR-7 and TLR-9, and proteinuria onset and anti-dsDNA IgG. (A-C) The relationship between **(A)** IgG deposits in glomeruli, as well as renal expression of **(B)** TLR-7 and **(C)** TLR-9, and proteinuria onset and anti-dsDNA IgG was analyzed by Spearman's rank correlation. This was determined in female mice fed either a low or high VD diet, with the latter containing 38,000 IU/kg. Graphed are numerical values, Spearman's rank correlation coefficient and its strength. $P \leq 0.05$ was considered significant, $p \geq 0.2$ is indicated as *ns, not significant*. Abbreviations: TLR = Toll-like receptor; VD = vitamin D.

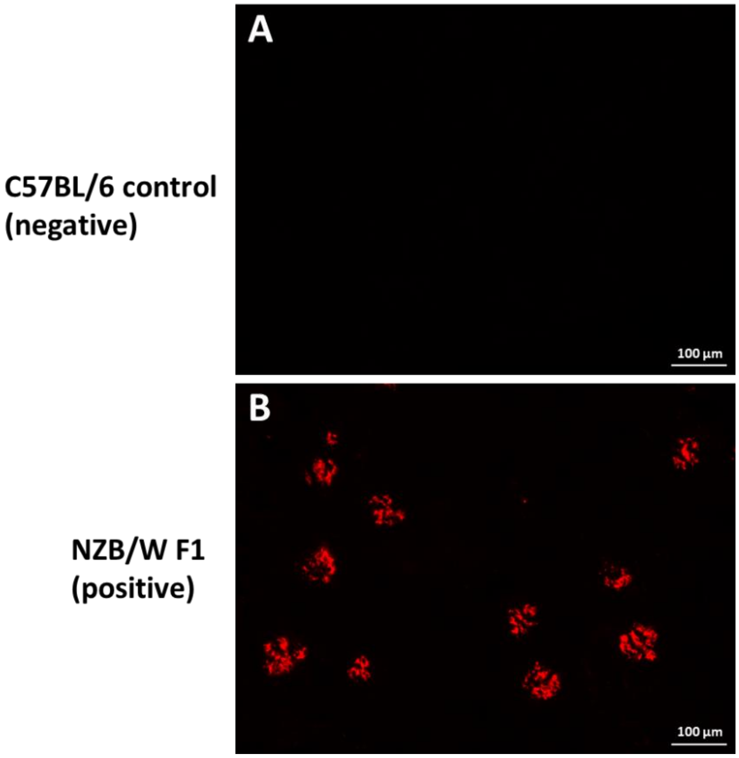

Supplementary Figure 4: Immunofluorescence for renal IgG deposits. Immunofluorescence for IgG shows negative staining in kidney of **(A)** C57BL/6 control mice and positive staining in kidney of **(B)** 25-26-week-old female NZB/W F1 mice. Circular tissue regions displaying high fluorescence represent individual glomeruli with IgG deposits.

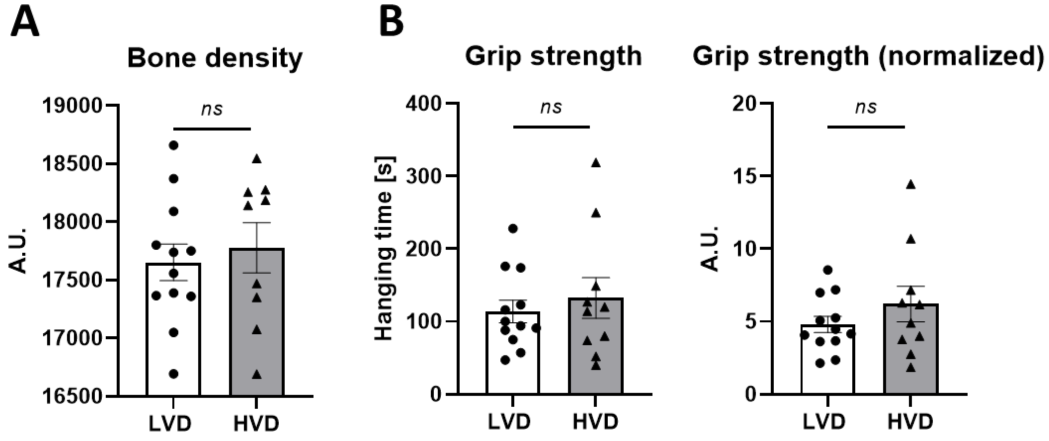

Supplementary Figure 5: Effects of low vitamin D intake on bone density and grip strength in female NZB/W F1 mice. (A) Bone density of 25-week-old female NZB/W F1 mice fed either a low VD or high VD diet. **(B)** Grip strength of 25-week-old female NZB/W F1 mice fed either a low VD or high VD diet, defined as inverted hanging time, or inverted hanging time, normalized to bodyweight. Results are displayed as scatter plots, with each data point representing one individual mouse. Data are expressed as mean ± SEM. $P \leq 0.05$ was considered significant, $p \geq 0.2$ is indicated as *ns, not significant*. N/group: LVD = 12; HVD = 9-10. Abbreviations: A.U. = arbitrary units; LVD = low vitamin D; HVD = high vitamin D.

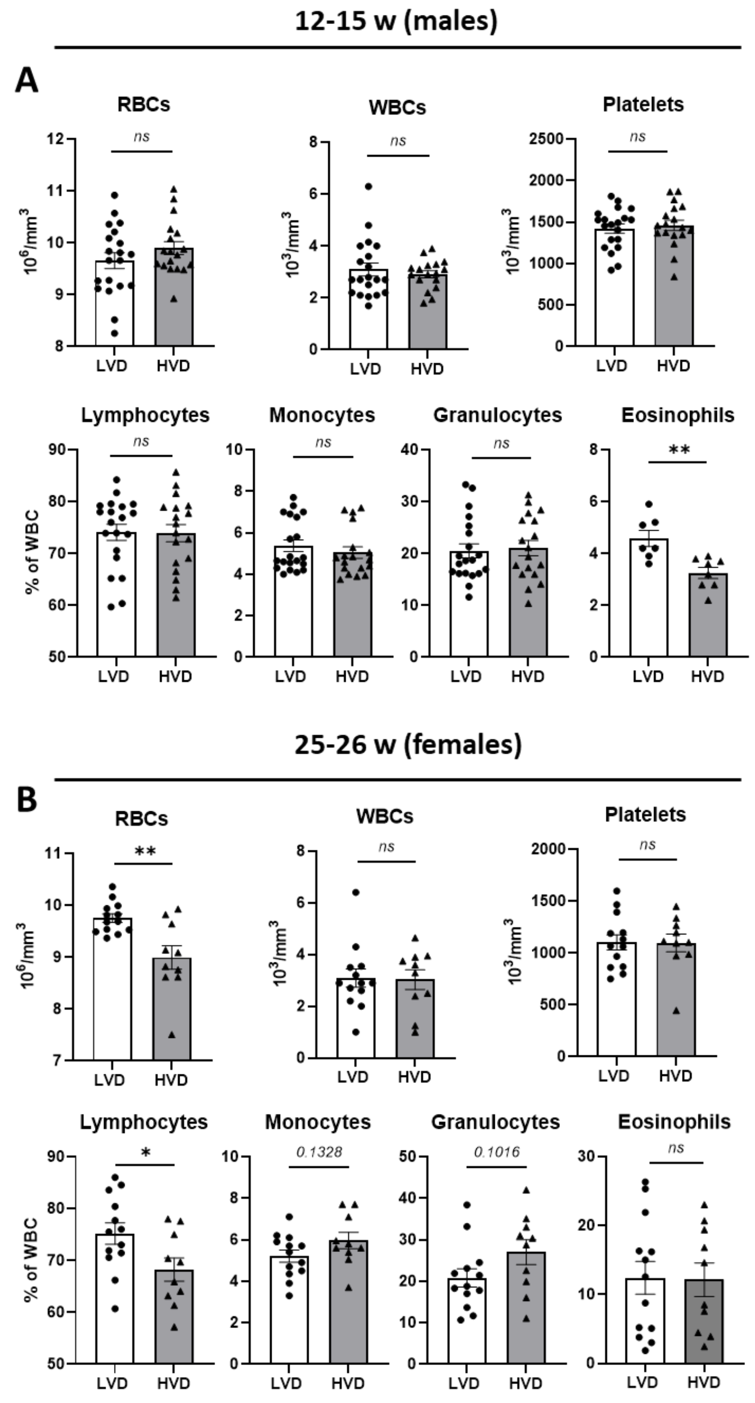

Supplementary Figure 6: Impact of low vitamin D intake on the distribution of blood cell populations (A, B) Hematology analysis of red blood cells (RBCs), white blood cells (WBCs) and platelets, as well as WBC subsets, in peripheral blood of **(A)** 12-15-week-old male and **(B)** 25-26-week-old LVD and HVD female NZB/W F1 mice. Results are displayed as scatter plots, with each data point representing an individual mouse. Data are expressed as mean ± SEM. $P \leq 0.05$ was considered significant, $p \geq 0.2$ is indicated as *ns, not significant*. N/group: males = 7-20; females = 10-13. Abbreviations: Tregs = regulatory T cells; LVD = low vitamin D; HVD = high vitamin D.

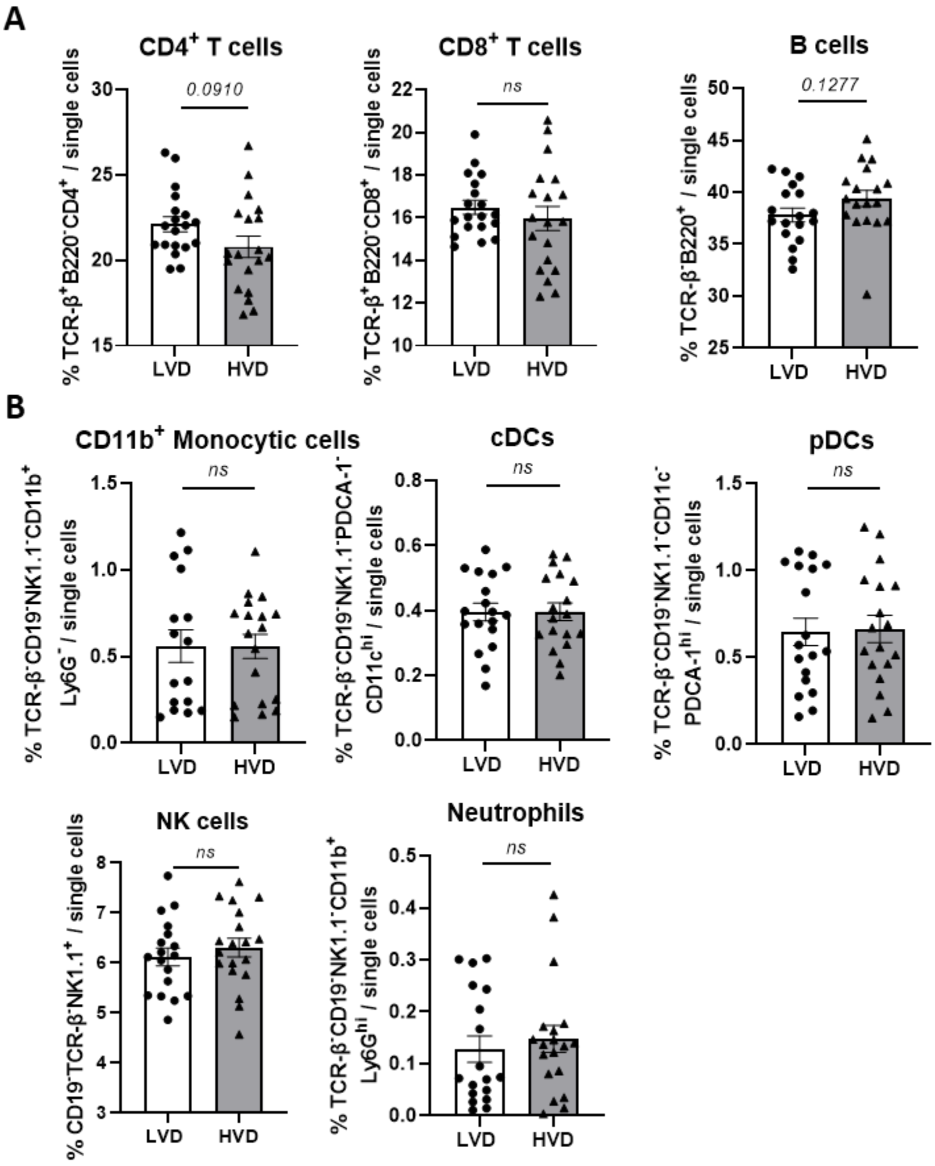

Supplementary Figure 7: Impact of low vitamin D intake on the distribution of main adaptive and innate immune cell populations in the spleen of male NZB/W F1 mice. (A, B) Frequencies of the main **(A)** adaptive and **(B)** innate immune cell populations in the spleen of 12-15-week-old LVD (n = 16-18) and HVD (n = 18-19) male NZB/W F1 mice. Results are displayed as scatter plots, with each data point representing an individual mouse. Data are expressed as mean ± SEM. $P \leq 0.05$ was considered significant, $p \geq 0.2$ is indicated as *ns, not significant*. Abbreviations: cDC = conventional dendritic cell; pDC = plasmacytoid dendritic cell; NK = natural killer; LVD = low vitamin D; HVD = high vitamin D.

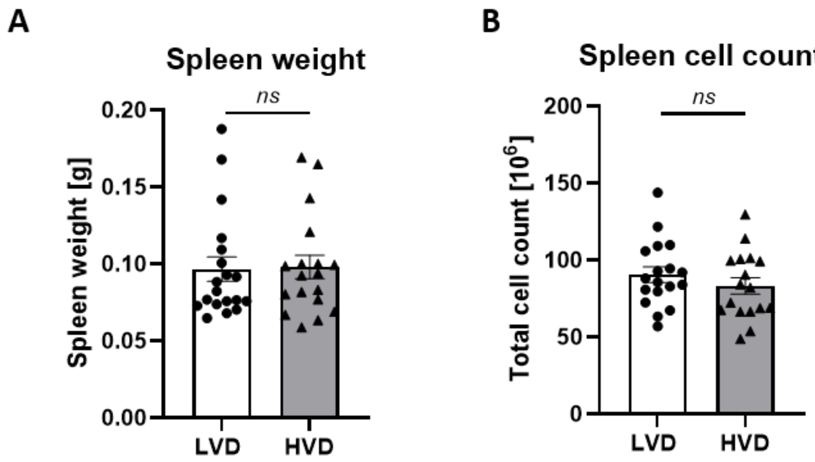

Supplementary Figure 8: Spleen weight and spleen cell counts of male NZB/W F1 mice. (A) Spleen weights of 12-15-week-old male NZB/W F1 animals. **(B)** Total spleen cell counts of 12-15-week-old male NZB/W F1 animals. Results are displayed as scatter plots, with each data point representing an individual mouse. Data are expressed as mean ± SEM. $P \leq 0.05$ was considered significant, $P \geq 0.2$ is indicated as *ns*, *not significant*. N/group: LVD = 18-19; HVD = 17-18. Abbreviations: LVD = low vitamin D; HVD = high vitamin D.

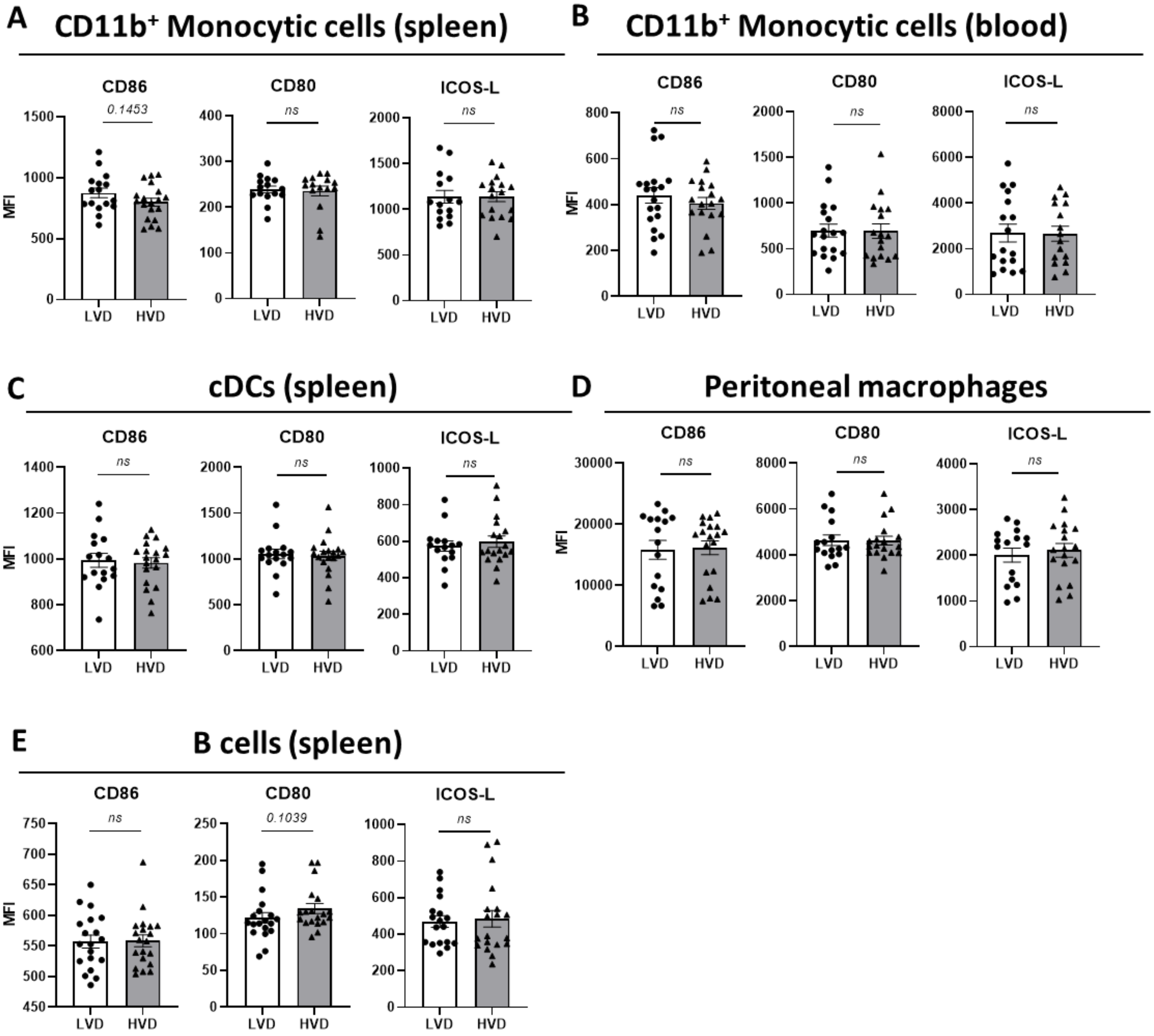

Supplementary Figure 9: Expression of co-stimulatory molecules on various immune cell subsets in male NZB/W F1 mice. (A-E) Surface expression of CD86, CD80 and ICOS-L was measured via flow cytometry on **(A)** splenic and **(B)** circulating $CD11b^+$ monocytic cells, **(C)** splenic conventional dendritic cells (cDCs), **(D)** peritoneal macrophages and **(E)** splenic B cells of 12-15-week-old male NZB/W F1 mice. Results are displayed as scatter plots, with each data point representing an individual mouse. Data are expressed as mean ± SEM. $P \leq 0.05$ was considered significant, $p \geq 0.2$ is indicated as *ns, not significant*. N/group: LVD = 15-19; HVD = 15-20. Abbreviations: MFI = mean fluorescence intensity; LVD = low vitamin D; HVD = high vitamin D.

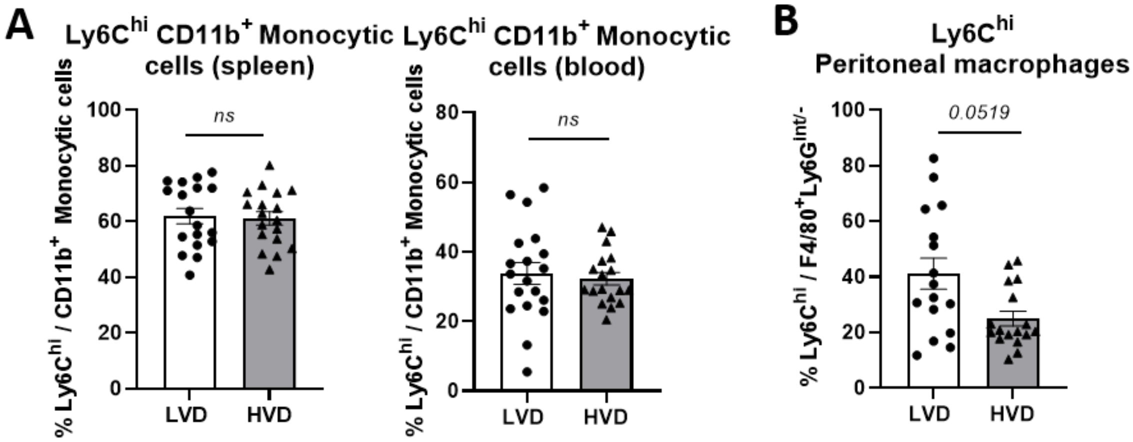

Supplementary Figure 10: Frequencies of Ly6C^{hi} CD11b^{+} monocytic cells and Ly6C^{hi} peritoneal macrophages. (A-B) The frequencies of Ly6C^{hi} **(A)** splenic and circulating CD11b^{+} monocytic cells, as well as **(B)** peritoneal macrophages, were determined via flow cytometry in 12-15-week old male mice. Results are displayed as scatter plots, with each data point representing an individual mouse. Data are expressed as mean ± SEM. $P \leq 0.05$ was considered significant, $p \geq 0.2$ is indicated as *ns*, *not significant*. N/group: LVD = 16-19; HVD = 17-18. Abbreviations: LVD = low vitamin D; HVD = high vitamin D.

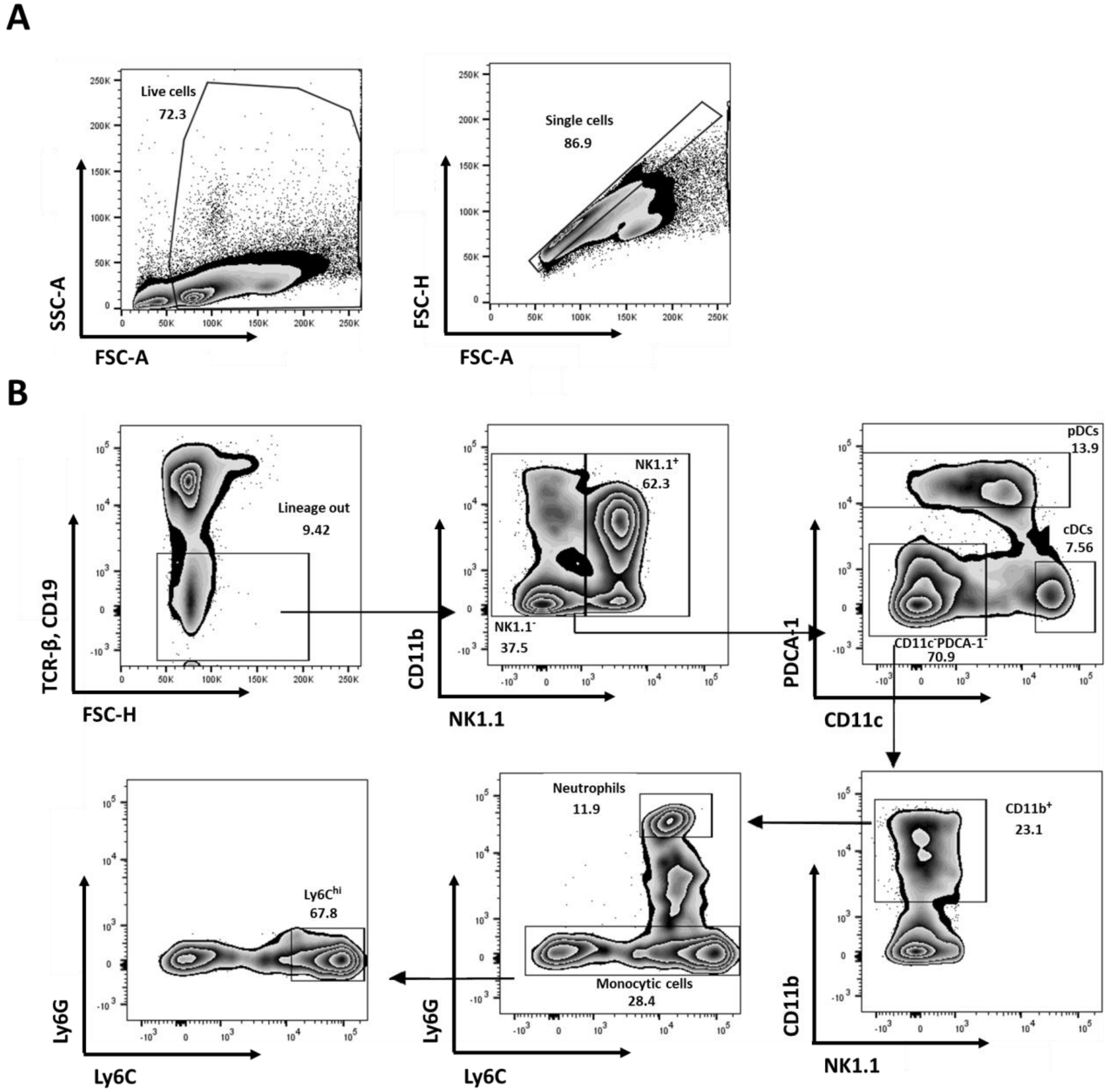

Supplementary Figure 11: Exemplary gating strategy for the selection of various innate immune cell populations in the spleen of NZB/W F1 mice. (A) For spleen cells, SSC-A was plotted against FSC-A for gating of live cells and FSC-H against FSC-A for exclusion of doublets. **(B)** Starting from single cells from the spleen, cells negative for defined lineage (Lin) markers (TCR-β^-CD19$^-$) were selected. NK cells were defined as Lin$^-$NK1.1$^+$, plasmacytoid dendritic cells (pDCs) as Lin-NK1.1-CD11c$^-$PDCA-1^{hi}, conventional dendritic cells (cDCs) as Lin$^-$NK1.1$^-$PDCA-1$^-$CD11c^{hi}, neutrophils as Lin$^-$NK1.1$^-$PDCA-1$^-$CD11c$^-$CD11b$^+$Ly6G^{hi} and CD11b$^+$ monocytic cells as Lin$^-$NK1.1$^-$PDCA-1^{lo}CD11c$^-$CD11b$^+$Ly6G$^-$. Further, Ly6C^{hi}CD11b+ monocytic cells were identified. Shown here are zebra plots originating from one representative donor.

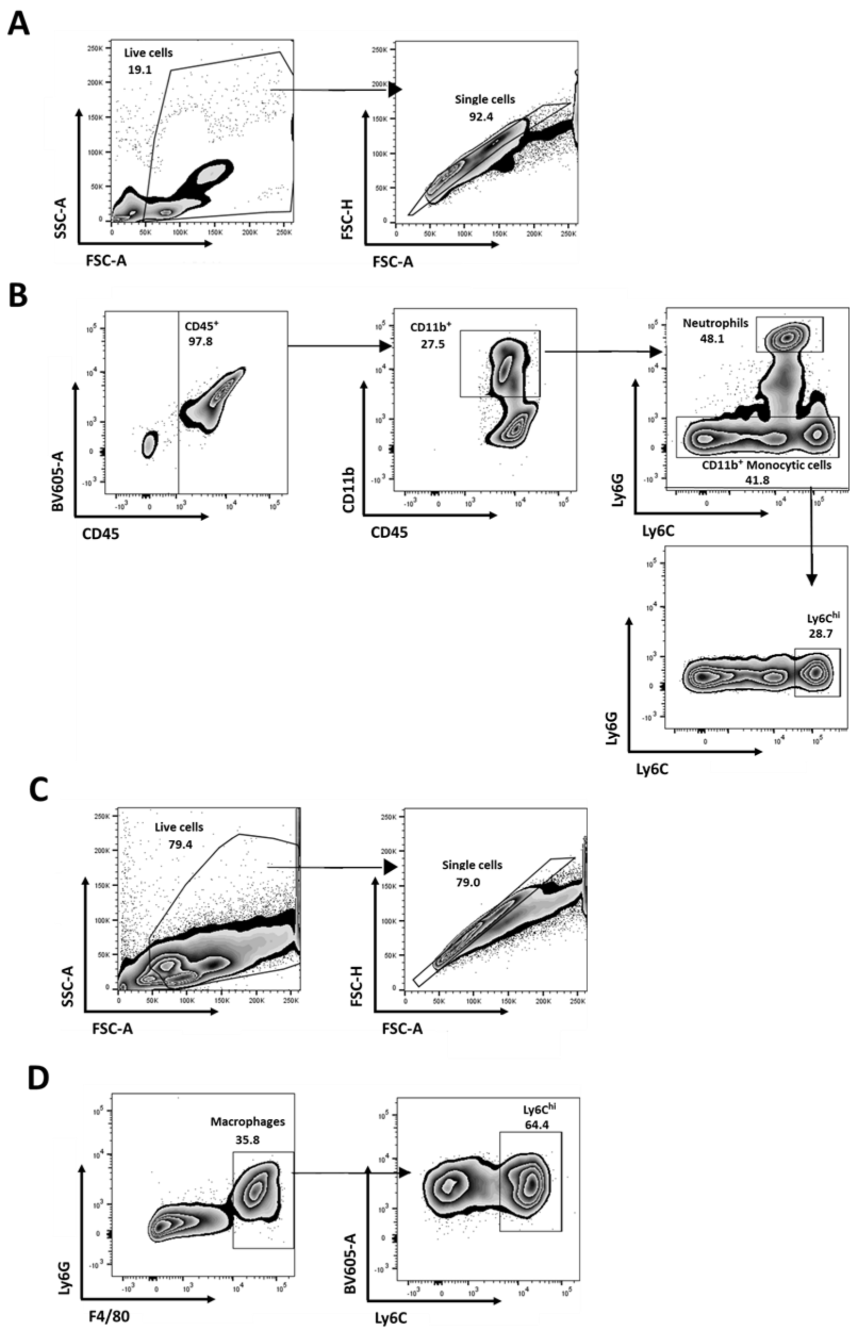

Supplementary Figure 12: Exemplary gating strategy for the selection of circulating innate immune cell populations and peritoneal macrophages in NZB/W F1 mice. (A) For peripheral blood cells, SSC-A was plotted against FSC-A for gating of live cells and FSC-H against FSC-A for exclusion of doublets. **(B)** Starting from single cells from blood, leukocytes were selected as $CD45^{+}$ cells. $CD11b^{+}$ leukocytes were selected and divided into neutrophils ($CD45^{+}CD11b^{+}Ly6G^{hi}$) and $CD11b^{+}$ monocytic cells ($CD45^{+}CD11b^{+}Ly6G^{-}$). Further, $Ly6c^{hi}CD11b^{+}$ monocytic cells were identified. **(C)** For peritoneal exudate cells (PECs), SSC-A was plotted against FSC-A for gating of live cells and FSC-H against FSC-A for exclusion of doublets. **(D)** Starting from single cells from PECs collected via peritoneal lavage, peritoneal macrophages were selected as $F4/80^{+}Ly6G^{int/-}$ cells. Further, $Ly6C^{hi}$ macrophages were identified. Shown here are zebra plots originating from one representative donor.

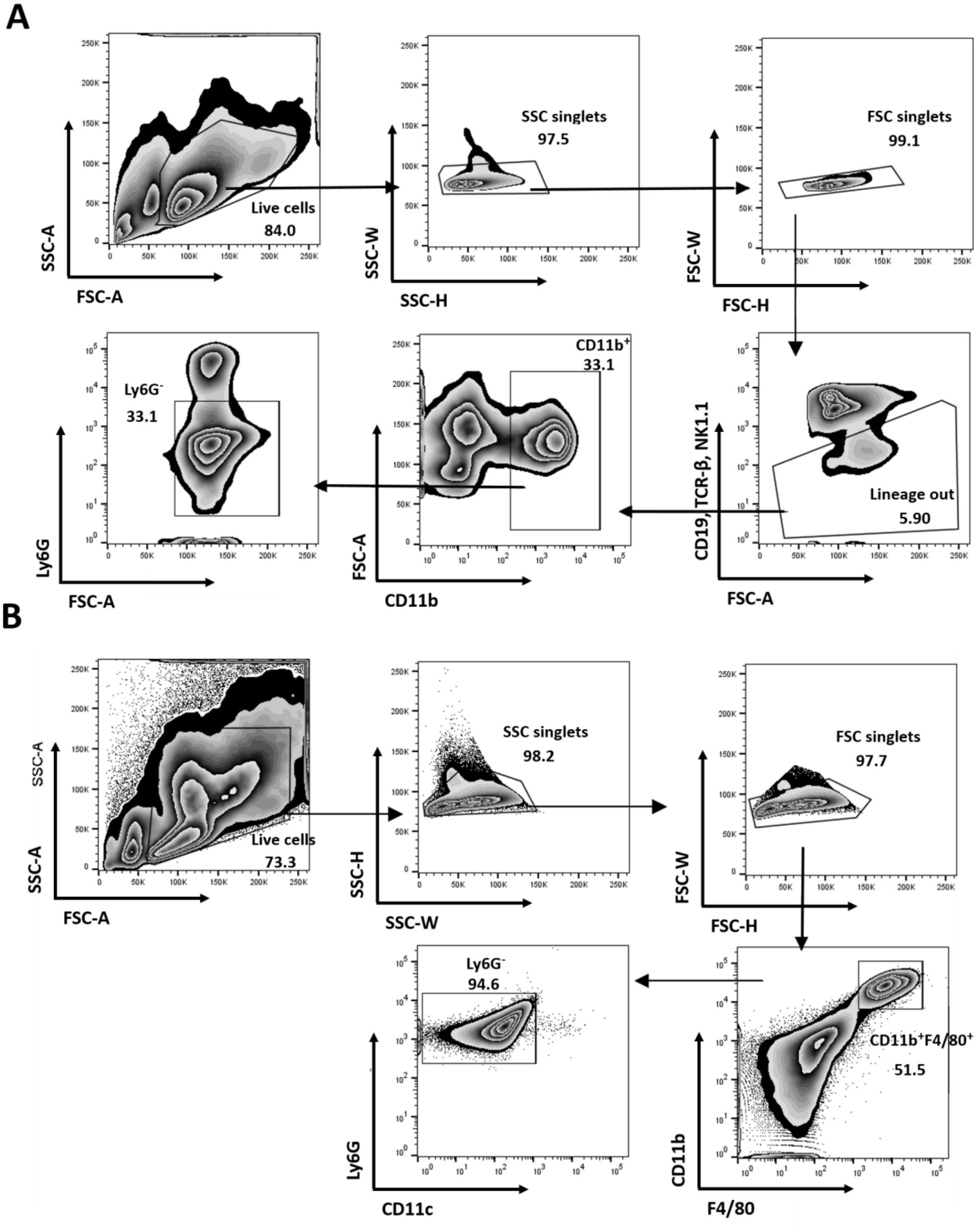

Supplementary Figure 13: Exemplary gating strategy for fluorescence-activated cell sorting of splenic CD11b$^+$ innate immune cells and peritoneal macrophages of NZB/W F1 mice. (A, B) SSC-A was plotted against FSC-A for gating of live cells and SSC-W against SSC-H as well as FSC-H against FSC-W for exclusion of doublets. **(A)** Splenic CD11b$^+$ innate immune cells were sorted as Lin$^-$ (CD19$^-$TCR-β$^-$NK1.1$^-$)CD11b$^+$Ly6G$^-$cells, while **(B)** peritoneal macrophages were sorted as F4/80$^+$CD11b$^+$CD11c$^-$Ly6G$^-$ cells after peritoneal lavage. Shown here are zebra plots originating from one representative donor.

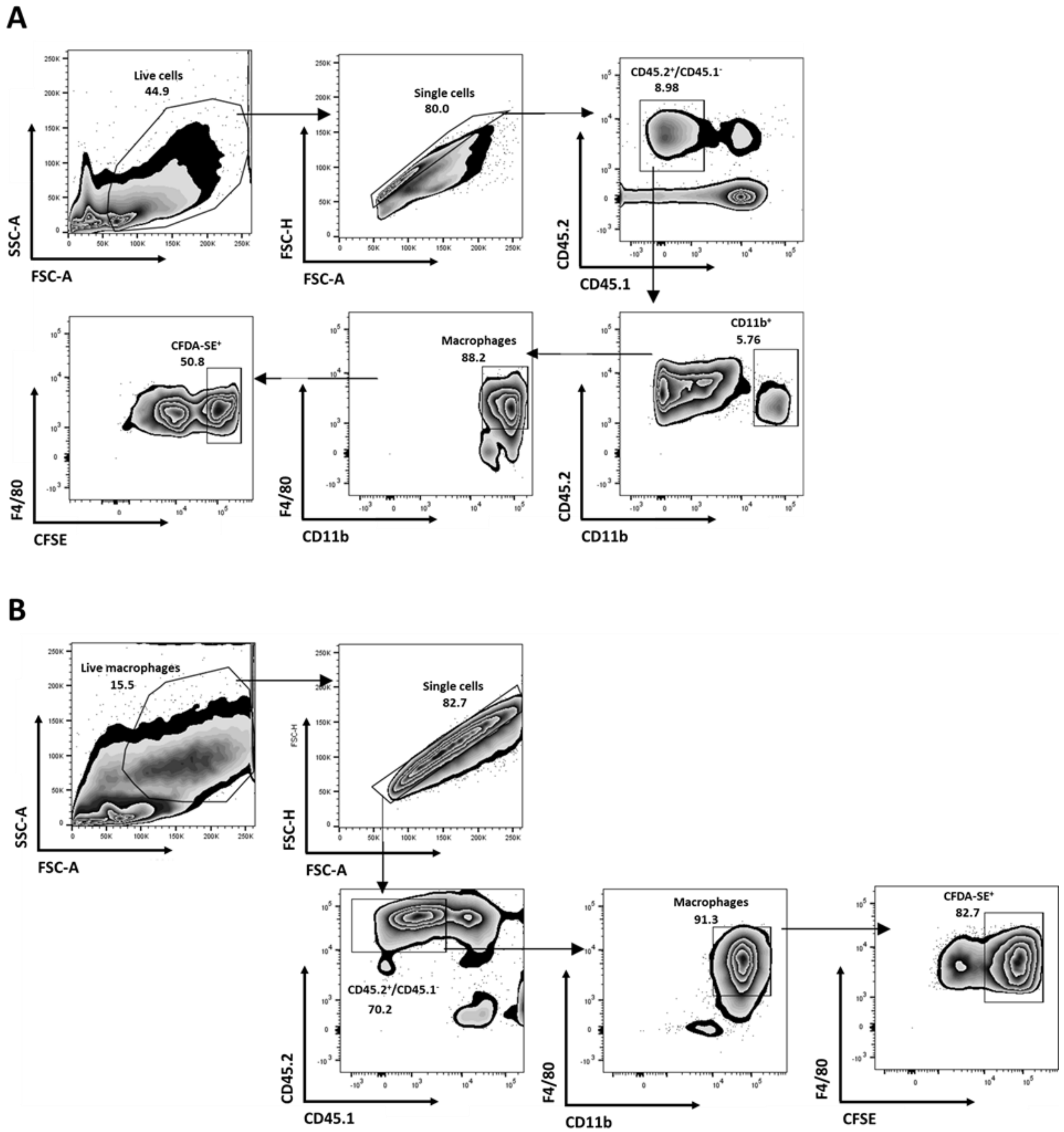

Supplementary Figure 14: Exemplary gating strategy for phagocytosis assays including peritoneal macrophages and BMDMs of NZB/W F1 mice. (A, B) SSC-A was plotted against FSC-A for gating of live cells and FSC-H against FSC-A for exclusion of doublets. $CD45.2^{+}CD45.1^{-}CD11b^{+}F4/80^{+}CFDA\text{-}SE^{+}$ **(A)** peritoneal macrophages and **(B)** BMDMs were defined as phagocytes that have phagocytosed apoptotic thymocytes (ATs). Shown here are zebra plots originating from one representative donor.

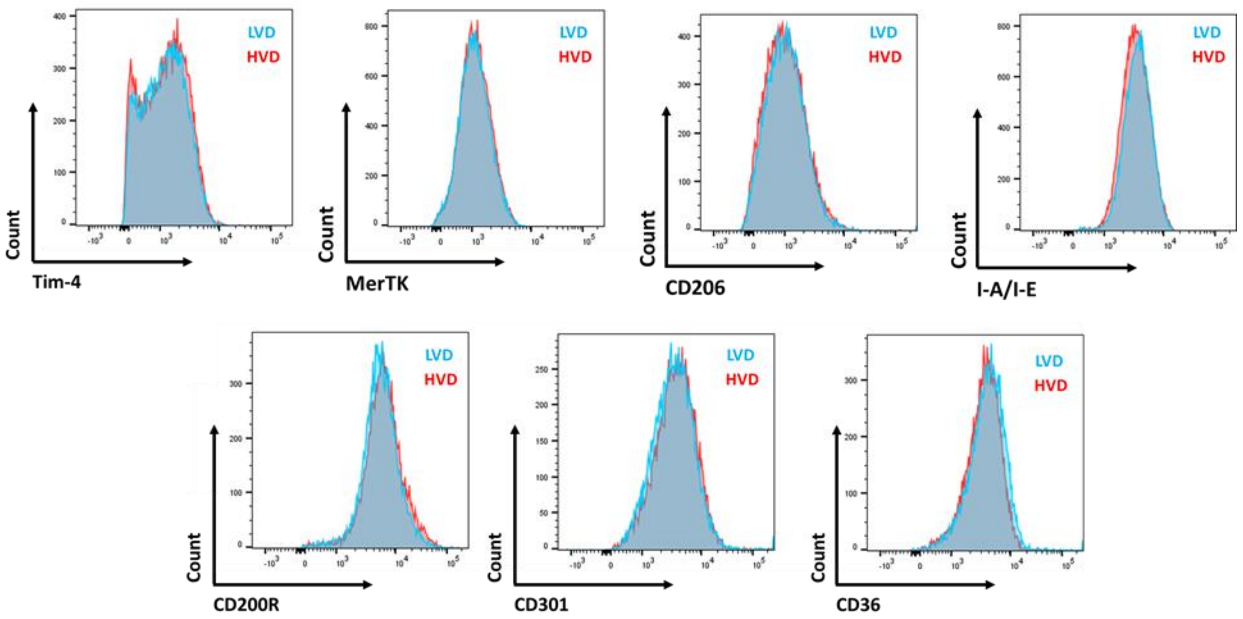

Supplementary Figure 15: Exemplary histograms of the surface expression of phagocytosis-associated markers. The surface expression of phagocytosis-associated markers was determined in bone-marrow-derived macrophages (BMDMs) (shown in figure) and peritoneal macrophages (not shown in figure). Depicted here are histograms originating from one representative donor of each treatment group (LVD and HVD). Abbreviations: LVD = low vitamin D; HVD = high vitamin D.

Supplementary Tables

Supplementary Table 1. Custom-designed primers used in qPCR analysis.

**Primer**	**Primer sequence (5’ → 3’)**
HPRT forward	GTTAAGCAGTACAGCCCCAAA
HPRT reverse	AGGGCATATCCAACAACAAACTT
TLR7 forward	GGCTGAACCATCTGGAAGAA
TLR7 reverse	TAAGCTGGATGGCAGATCCT
TLR9 forward	TATCCACCACCTGCACAACT
TLR9 reverse	TTCAGCTCCTCCAGTGTACG

Supplementary Table 2. All correlations between serum VD levels and survival, proteinuria onset and anti-dsDNA IgG.

Serum 25(OH)D_3 correlations	r	*P-value*
**All treatment groups (LVD, NVD, HVD 76,5000 IU/kg)**		
Serum 25(OH)D_3 – Overall survival	0.2012	*0.1751*
Serum 25(OH)D_3 – Proteinuria onset	0.3410	*0.0203**
Serum 25(OH)D_3 – Serum anti-dsDNA IgG	-0.3778	*0.0232**
**Individual treatment groups (LVD, NVD, HVD 76,5000 IU/kg)**		
LVD: Serum 25(OH)D_3 – Overall survival	-0.3767	*0.1839*
LVD: Serum 25(OH)D_3 – Proteinuria onset	0.2755	*ns*
LVD: Serum 25(OH)D_3 – Serum anti-dsDNA IgG	-0.0839	*ns*
NVD: Serum 25(OH)D_3 – Overall survival	-0.0325	*ns*
NVD: Serum 25(OH)D_3 – Proteinuria onset	0.3661	*0.1268*
NVD: Serum 25(OH)D_3 – Serum anti-dsDNA IgG	0.1909	*ns*
HVD: Serum 25(OH)D_3 – Overall survival	-0.2281	*ns*
HVD: Serum 25(OH)D_3 – Proteinuria onset	-0.1046	*ns*
HVD: Serum 25(OH)D_3 – Serum anti-dsDNA IgG	0.2802	*ns*
**All treatment groups (LVD, HVD 38,000 IU/kg)**		
Serum 25(OH)D_3 – Overall survival	0.4007	*0.0581*
Serum 25(OH)D_3 – Proteinuria onset	0.5145	*0.0101**
Serum 25(OH)D_3 – Serum anti-dsDNA IgG	-0.5892	*0.0049***
**Individual treatment groups (LVD, HVD 38,000 IU/kg)**		
LVD: Serum 25(OH)D_3 – Overall survival	-	-
LVD: Serum 25(OH)D_3 – Proteinuria onset	-	-
LVD: Serum 25(OH)D_3 – Serum anti-dsDNA IgG	-	-
HVD: Serum 25(OH)D_3 – Overall survival	0.0830	*ns*
HVD: Serum 25(OH)D_3 – Proteinuria onset	-0.1925	*ns*
HVD: Serum 25(OH)D_3 – Serum anti-dsDNA IgG	-0.2079	*ns*

The relationship between serum 25(OH)D_3 concentrations (20 w) and overall survival, proteinuria onset and serum anti-dsDNA IgG titers (20 w) was analyzed by Spearman's rank correlation. This was determined in an experiment where female mice were fed either a low, normal or high vitamin D (VD) diet, with the latter containing 76,500 IU/kg, as well as an experiment where female mice were fed either a low or high VD diet, with the latter containing 38,000 IU/kg. $P \leq 0.05$ was considered significant, $p \geq 0.2$ is indicated as *ns*, *not significant*. Abbreviations: LVD = low vitamin D; HVD = high vitamin D.

Supplementary Table 3. Innate immune changes in peripheral blood and peritoneal macrophages of LVD and HVD mice.

	12-15 w (males)			25-26 w (females)		
**Peripheral blood**	**LVD** **Mean +/- SEM**	**HVD** **Mean +/- SEM**	***P-value***	**LVD** **Mean +/- SEM**	**HVD** **Mean +/- SEM**	***P-value***
**Distribution of immune cell populations**						
**Neutrophils:** $CD11b^{+}Ly6G^{hi}$ / $CD45^{+}$ cells [%]	16.89 +/- 1.86	16.14 +/- 1.76	*ns*	12.40 +/-1.06	16.43 +/- 1.48	*0.0148**
**$CD11b^{+}$ Monocytic cells:** $CD11b^{+}Ly6G^{-}$ / $CD45^{+}$ cells [%]	14.24 +/- 0.90	14.11 +/- 0.72	*ns*	17.53 +/- 1.73	23.63 +/- 3.65	*0.1071*
**$Ly6C^{hi}$ $CD11b^{+}$ Monocytic cells:** $Ly6C^{hi}$ / $CD11b^{+}$ Monocytic cells [%]	33.79 +/- 3.16	32.3 +/- 1.75	*ns*	22.08 +/- 1.62	20.48 +/- 1.97	*ns*
**Co-stimulatory molecules**						
Total $CD11b^{+}$ monocytic cells:						
CD86 MFI	441.00 +/- 33.38	405.89 +/- 25.09	*ns*			
CD80 MFI	699.61 +/- 69.30	695.35 +/- 76.55	*ns*			
ICOS-L MFI	2690.5 +/- 377.9	2661.1 +/- 320.1	*ns*			
**Peritoneal macrophages**						
**Distribution of cell populations**						
**$Ly6C^{hi}$ Peritoneal macrophages:** $Ly6C^{hi}$ / Peritoneal macrophages [%]	41.11 +/- 5.39	24.95 +/- 2.56	*0.0519*			
**Co-stimulatory molecules**						
Total peritoneal macrophages						
CD86 MFI	15819 +/- 1491.1	16157 +/- 4802.8	*ns*			
CD80 MFI	4637.9 +/- 206.1	4619 +/- 186.7	*ns*			
ICOS-L MFI	2001.2 +/- 136.4	2112.7 +/- 145.2	*ns*			

Flow cytometry analysis of peripheral blood and peritoneal macrophages of 12-15-week-old LVD and HVD male, as well as 25-26-week-old LVD and HVD female NZB/W F1 mice. Results are expressed as mean +/- SEM. N/group: males = 17-19; females = 10-13. $P \leq 0.05$ was considered significant, $p \geq 0.2$ is indicated as *ns*, *not significant*. Abbreviations: LVD = low vitamin D; HVD = high vitamin D.
